# Revisiting the Poison Dart Frog *Ameerega ingeri* (Anura: Dendrobatidae): external morphology, tadpoles, natural history, distribution, advertisement call, phylogenetic position and conservation

**DOI:** 10.7717/peerj.20078

**Published:** 2025-12-12

**Authors:** Juan C. Diaz-Ricaurte, Alejandro Navarro-Morales, César Malambo, Junner F. González-Ibarra, Yulfreiler Garavito-David, Felipe Silva de Andrade, Mario A. Madrid-Ordoñez, Maykoll J. Parra-Olarte, Diego Huseth Ruiz-Valderrama, Betselene Murcia-Ordoñez, Amanda Varago, Diego J. Santana

**Affiliations:** 1 Departamento de Ecologia, Instituto de Biociências, Universidade de São Paulo, São Paulo, São Paulo, Brazil; 2 Grupo de investigación en Biodiversidad y Desarrollo Amazónico (BYDA), Semillero de Investigación en Ecofisiología y Biogeografía de Vertebrados, Centro de investigaciones Amazónicas Macagual—César Augusto Estrada González, Universidad de la Amazonia, Florencia, Caquetá, Colombia; 3 Semillero de Investigación en Herpetología—SEH, Universidad de la Amazonia, Florencia, Caquetá, Colombia; 4 Centro de Investigación de la Biodiversidad Andino Amazónica - INBIANAM, Universidad de la Amazonia, Florencia, Caquetá, Colombia; 5 Institución Educativa Antonio Ricaurte, Florencia, Caquetá, Colombia; 6 Programa de Biología, Grupo de Investigación BYDA, Universidad de la Amazonia, Florencia, Caquetá, Colombia; 7 Laboratório de Evolução e Biologia Integrativa (LEBI), Departamento de Biologia, Faculdade de Filosofia, Ciências e Letras de Ribeirão Preto (FFCLRP), Universidade de São Paulo, Ribeirão Preto, São Paulo, Brazil; 8 Museu de Diversidade Biológica (MDBio), Instituto de Biologia (IB), Universidade Estadual de Campinas, Campinas, São Paulo, Brazil; 9 Parque Nacional Natural Alto Fragua-Indi Wasi, San José del Fragua, Caquetá, Colombia; 10 Conservación Internacional Colombia, Programa del Corredor Trasandino Amazónico de Colombia, Bogota D.C., Colombia; 11 Grupo de investigación en Biodiversidad y Desarrollo Amazónico (BYDA), Centro de investigaciones Amazónicas Macagual—César Augusto Estrada González, Universidad de la Amazonia, Florencia, Caquetá, Colombia; 12 Instituto de Biociências, Universidade Federal de Mato Grosso do Sul, Campo Grande, Mato Grosso do Sul, Brazil

**Keywords:** Amphibian conservation, Dendrobatidae, Natural history, Species redescription, Geographical distribution, DNA, Phylogenetic relationships, Taxonomy, Bioacustics, Species identification

## Abstract

Many amphibian species have been described based solely on preserved specimens, leaving significant gaps in our understanding of their behavior, natural history, population status, and phylogenetic relationships—factors essential for effective conservation. *Ameerega ingeri*, a dark poison frog originally believed to be endemic to Colombia, exemplifies this challenge. Described from only four specimens, its coloration in life, ecological traits, and distribution remained poorly understood for decades, leading to taxonomic confusion with closely related species. In this study, we revisit *A. ingeri* by providing new distribution records that extend its known range, along with detailed morphological and acoustic descriptions, dietary analysis, and population data. By integrating molecular and acoustic data with ecological and natural history observations, we clarify its taxonomic identity and distinguish it from congeners. Furthermore, we assess its conservation status based on updated occurrence and population data, and propose its inclusion in a threat category according to IUCN criteria. Our findings enhance the understanding of dendrobatid diversity and highlight the importance of field-based research on poorly known and potentially threatened species to support informed conservation strategies.

## Introduction

The Amazon ecoregions are recognized for their exceptional terrestrial biodiversity (Hoorn et al., 2010; Guayasamin et al., 2024), particularly for amphibians, with approximately 947 species (Penhacek et al., 2024). This rich diversity is driven by a variety of habitats shaped by geological, hydrological, and climatic factors (Barrera et al., 2007). However, the knowledge on amphibian species from the Andean-Amazon piedmont and Colombian Amazon remains limited (Acosta-Galvis, 2000; Ruiz et al., 2007). This scarcity is primarily due to challenges such as inaccessible and unexplored areas requiring complex and expensive logistics, further exacerbated by the historical presence of armed groups such as the FARC (Fuerzas Armadas Revolucionarias de Colombia) (Etter et al., 2006; Reardon, 2018), which hindered scientific exploration. Additionally, while extensive deforestation and livestock farming from unsustainable agriculture have severely impacted the region’s biodiversity, reducing the species richness present, the inaccessibility and security concerns are the primary factors directly limiting data collection efforts. These factors are particularly pronounced in the department of Caquetá (Capdevilla, Bermúdez & Aguirre, 2023), located in the south of Colombia.

Despite the geographic and social challenges such as the complex and expensive logistics to access unexplored areas, this department harbors remarkable diversity of anurans (Acosta-Galvis, 2000; Galeano et al., 2006; Pérez, Velásquez & Castro, 2012; Acosta-Galvis, 2025). Among the 204 species recorded in this region, 11 belong to the family Dendrobatidae, commonly known as poison frogs (Acosta-Galvis, 2025; Frost, 2025). This family is distributed from high mountain forests over the Andes to floodplain forests in the Amazon, exhibiting semi-aquatic, terrestrial, and arboreal habits (Lötters et al., 2007; Almendáriz, Ron & Brito, 2012). Their reproductive behaviors occur in leaf litter, ponds, and small bodies of water found in terrestrial and epiphytic plants (Summers & McKeon, 2004; Summers, McKeon & Heying, 2006). Additionally, this group displays parental care, including transporting tadpoles on their backs and provisioning them with oocytes as food (Myers & Daly, 1983; Grant et al., 2006; Valderrama-Vernaza, Ramírez-Pinilla & Serrano-Cardozo, 2009; Ringler et al., 2017; O’Connell, 2020). Dendrobatidae exhibits a wide range of shades, intensities, and color patterns (Summers & Clough, 2001), which often results in overlaps in morphology among species (Everman & Wang, 2019). This variability poses challenges for taxonomic identification, as the morphological traits used tend to differentiate species groups rather than individual species, leading to ambiguous taxonomic statuses (Stockman & Bond, 2007). This is particularly evident in the genus *Ameerega* (Twomey & Brown, 2008).

This genus comprises 29 described species with a strictly cis-Andean distribution, ranging from Mato Grosso do Sul and Goiás in Brazil, extending northward and northwestward through the Amazon to the Andean foothills in Bolivia and Venezuela (Frost, 2025). Among the five *Ameerega* species found in Colombia, *A. ingeri* (Cochran & Goin, 1970) is known from the departments of Caquetá (Cochran & Goin, 1970; Silverstone, 1976; Ruiz-Carranza, Ardila-Robayo & Lynch, 1996; Acosta-Galvis, 2000; Lötters et al., 2007; Malambo, 2016; Gutiérrez et al., 2020) and Putumayo (Guerrero-Vargas et al., 2007), at elevations ranging from 100 to 1,191 m (Acosta-Galvis, 2025).

*Ameerega ingeri* was originally described based on one holotype and three paratypes collected from Aserrío, near Río Pescado in the Caquetá department of Colombia, at an elevation of approximately 300 m (Cochran & Goin, 1970). Initially, this species was misidentified as *Epipedobates pictus* and later synonymized with *A. bilinguis* (see Amezquita, Rueda-Almonacid & Rueda-Martínez, 2004). Subsequently, it was placed within the *A. picta* group (Lötters et al., 2007). Silverstone (1976) provided a morphological characterization of *Ameerega ingeri*, based on preserved specimens. According to the author, the holotype is a 27.0 mm female, with the skin strongly granular on the dorsum and dorsal surfaces of the hind limbs, and smooth on the forelimbs, venter, and ventral surfaces of the hind limbs. Maxillary and premaxillary teeth are present, and the first finger is longer than the second. The toes are webless. The coloration in life was unknown at the time, but in preservative, the species is entirely black, lacking dorsolateral and lateral stripes. White spots are present on the axilla and the proximodorsal surface of the upper arm, the proximodorsal surface of the thigh (either round or rectangular), and the proximoventral surface of the calf (sometimes extending halfway down the leg). Additionally, the holotype shows ill-defined gray marbling on the belly, while paratypes exhibit similar marbling on the venter and ventral surfaces of the limbs.

Recently, populations near the type locality were included in a comprehensive phylogeny of the genus (Guillory et al., 2020). These populations, referred to as *A. ingeri*, were recovered as the sister group to the clade containing all other *Ameerega* species except *A. silverstonei*. This suggests that *A. ingeri* does not have any close relatives within the genus and may represent a lineage from the genus’ initial diversification (Guillory et al., 2020). Given its rarity, and despite its recent inclusion in a phylogeny, detailed information on the morphology of adults and tadpoles, as well as data on their advertisement call, natural history, distribution, and population density, remains scarce. This study aims to address these knowledge gaps by revisiting the adult morphology and advertisement calls, and by providing new insights into the species’ natural history, diet, and distribution. Based on this integrative information, we also propose a preliminary conservation status assessment for the species.

## Material and Methods

### Study area

**Figure 1:**
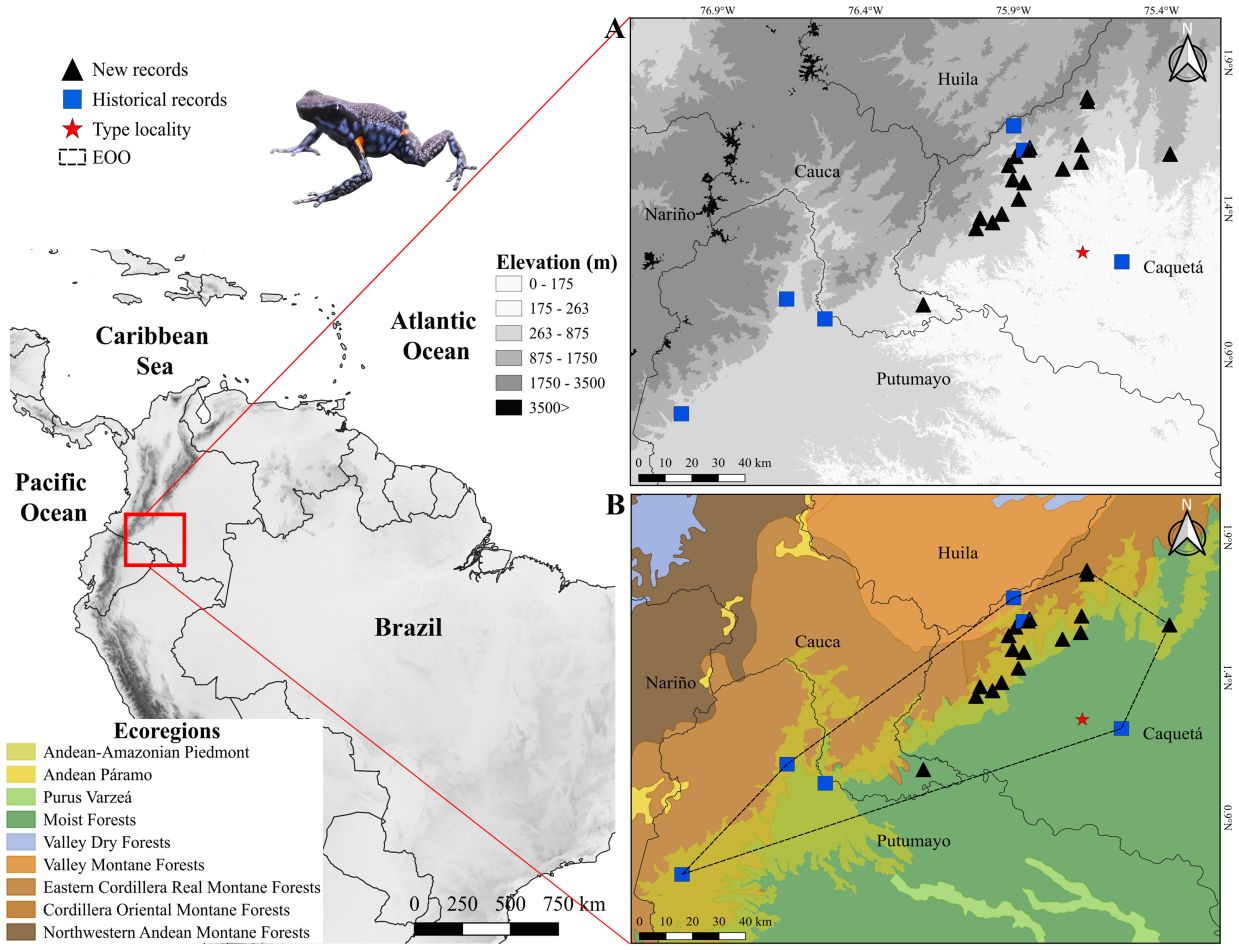
Distribution of *Ameerega ingeri*. Distribution of *Ameerega ingeri* (A) Elevation. (B) EOO (Extent of occurrence = 7,798.333 Km^2^) and upper bound of AOO (Area of occupancy = 92 Km^2^) with ecoregions from Dinerstein et al. (2017).

The study sites comprised secondary and gallery forests with some peripheral anthropogenic pressures, in areas embedded within the National System of Protected Areas, such as National Natural Parks and Natural Reserves of Civil Society, prioritizing conservation (RUNAP Registro Único Nacional de Áreas Protegidas, 2024). Located on the eastern slope of the eastern mountain range, within the Andean-Amazonian Piedmont of Caquetá department (300–1,000 m elevation), these sites are considered buffer zones between the eastern mountain range and the Amazon plain (Robertson & Castiblanco, 2011) (Fig. 1). This ecoregion exhibits an annual temperature of 26 °C, precipitation of 3,700 mm, and relative humidity between 69–86% (Peña Nuñez, Jiménez-Ferreira & Pasaje-Bolaños, 2017).

We recorded specimens of *A. ingeri* in 16 areas (seven localities) in the municipalities of San José del Fragua (SJF), Belén de los Andaquíes (BDA), Florencia and El Paujil, Department of Caquetá, Colombia (Fig. 1, Table S1). Specifically, the localities are: San José del Fragua (SJF; 1.352876 N, 75.971376 W; 477 m elevation), Parque Nacional Natural Alto Fragua-Indi Wasi (BDA-1; 1.579600 N, 75.893100 W; 971 m elevation), Finca El Diviso (BDA-2; 1.499586 N, 75.903042 W; 444 m elevation), Finca La Esperanza (BDA-3; 1.382000 N, 75.940400 W; 440 m elevation). All of these localities correspond to secondary forests with influence on crop and pasture areas. The localities in Florencia are: Reserva Natural y Ecoturística La Avispa (RLA; 1.618947 N, 75.669273 W; 432 m elevation) and Finca El Tigre (FET; 1.536444 N, 75.734056 W; 456 m elevation). The locality in El Paujil is the Reserva Natural y Agroturística Doña Blanca (RDB; 1.587139 N, 75.371194 W; 853 m elevation). The RLA and RDB are localities with conserved areas in secondary and gallery forests. Finally, the FET shows different land uses that are commonly dedicated to agricultural production with some areas of gallery and secondary forests (mostly in forest restoration and natural regeneration). For more details about the distances between the type locality and the localities reported in this work, please see the Supplementary Material.

### Fieldwork

Fieldwork was carried out between 2017 and 2022 at seven localities, during diurnal (07:00–12:00 h) and nocturnal (18:00–22:00 h) periods, employing unrestricted visual encounter surveys (VES; Angulo et al., 2006), thoroughly reviewing all available microhabitats such as rocks, cracks, bodies of water, fallen logs, leaf litter, among others (Ramírez-Bautista et al., 2010) with a daily sampling effort approximately 18h/person. Acoustic recordings of the individuals were made in 2024.

The collected specimens (39 specimens from the 15 localities) were euthanized and fixed in 10% formalin, and preserved in 70% ethanol, following the standard procedures proposed by McDiarmid (1994). Free-swimming tadpoles were collected, raised and preserved at several stages of development in 10% formalin proposed by (McDiarmid & Altig, 1999). The specimens collected under of license #2016018668-1-000; COL0071015 by Autoridad Nacional de Licencias Ambientales (ANLA) and deposited in the amphibian collection of the Museo de Historia Natural, Universidad de la Amazonia, Florencia, Caquetá, Colombia (UAM-H) (Table S1).

### Species identification and external morphology

Specimens were identified following the literature (Cochran & Goin, 1970; Silverstone, 1976). We examined high-resolution photographs of the holotype of *Ameerega ingeri* deposited at the Division of Amphibians & Reptiles Collections of Smithsonian National Museum of Natural History. These images allowed us to make direct comparisons between the type material and the newly analyzed specimens, supporting our identification and redescription.

**Figure 2:**
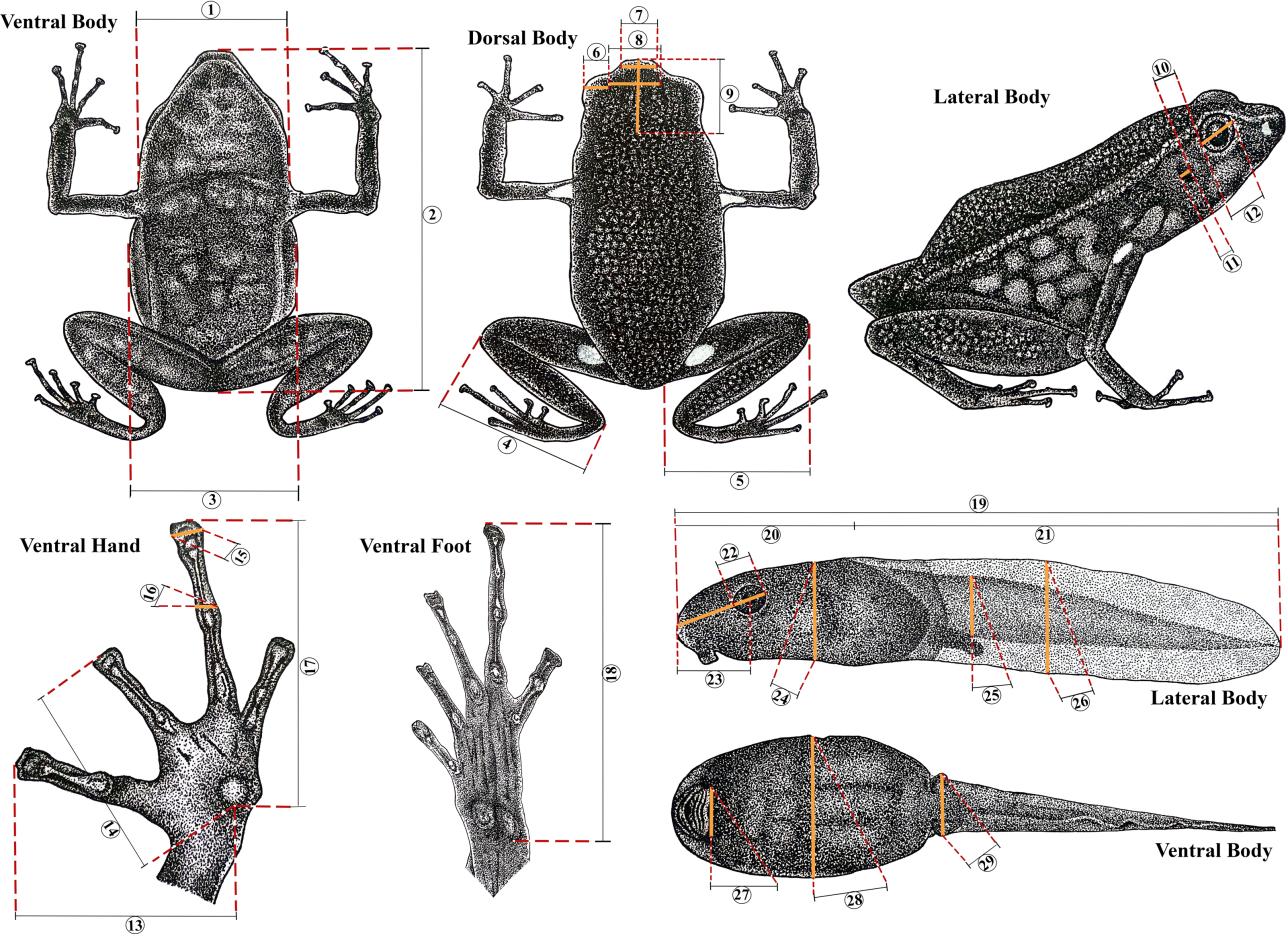
Morphometric measurements in *Ameerega ingeri*. Morphometric measurements of adult and tadpole stages of *Ameerega ingeri*. (1) HW. (2) SVL. (3) BW. (4) TL. (5) FL. (6) UEW. (7) IND. (8) IOD. (9) HL. (10) DET. (11) TD. (12) ED. (13) L1F. (14) L2F. (15) W3D. (16) W3F. (17) HaL. (18) FoL. (19) TL. (20) BL. (21) TAL. (22) ED. (23) END. (24) BH. (25) TMH. (26) MTH. (27) ODW. (28) BW. (29) TMW. Illustration by Yorlan Andres Repizo Perdomo.

The body dimensions were measured using a digital caliper (0.01 mm) and measurements are given only for adult individuals. Adults were sexed by observing their gonads, vocal slits and size. Measurements follow Myers (1982) and Twomey & Brown (2008): Snout-vent length (SVL), femur length from vent to lateral edge of knee (FL), tibia length from medial edge of heel to lateral edge of knee (TL), foot length from proximal edge of metatarsal tubercle to tip of toe IV (FoL), hand length from proximal edge of metacarpal tubercle to tip of longest finger (HaL), head length from most exposed corner of occipital to tip of snout (HL), head width between tympana (HW), body width under axillae (BW), upper eyelid width (UEW), interorbital distance (IOD), internarial distance (IND), horizontal tympanum diameter (TD), horizontal eye diameter (ED), distance from outer corner of eye to tympanum (DET), length of finger I from proximal edge of median palmar tubercle to tip of finger disc (L1F), length of finger II from proximal edge of median palmar tubercle to tip of finger disc (L2F), width of disc of finger III (W3D), and width of finger III just below disc (W3F) (Fig. 2).

Preserved tadpoles were staged according to Gosner (1960) and measured (millimeters) with an ocular micrometer in a dissecting stereomicroscope. The following measurements (Poelman et al., 2010) were taken for each tadpole: total length (TL), body length (BL), body height (BH), body width (BW), head width at level of the eyes (HW), eye diameter (ED), eye–naris distance (END), nostril–snout distance (NSD), internarial distance (IND), interorbital distance (IOD), tail length (TAL), maximum height of tail (MTH), tail muscle height at base of tail (TMH), tail muscle width at base of tail (TMW) and oral disc width (ODW). Mouthpart formulae and terminology follow McDiarmid & Altig (1999).

### Natural history and ecology

**Figure 3:**
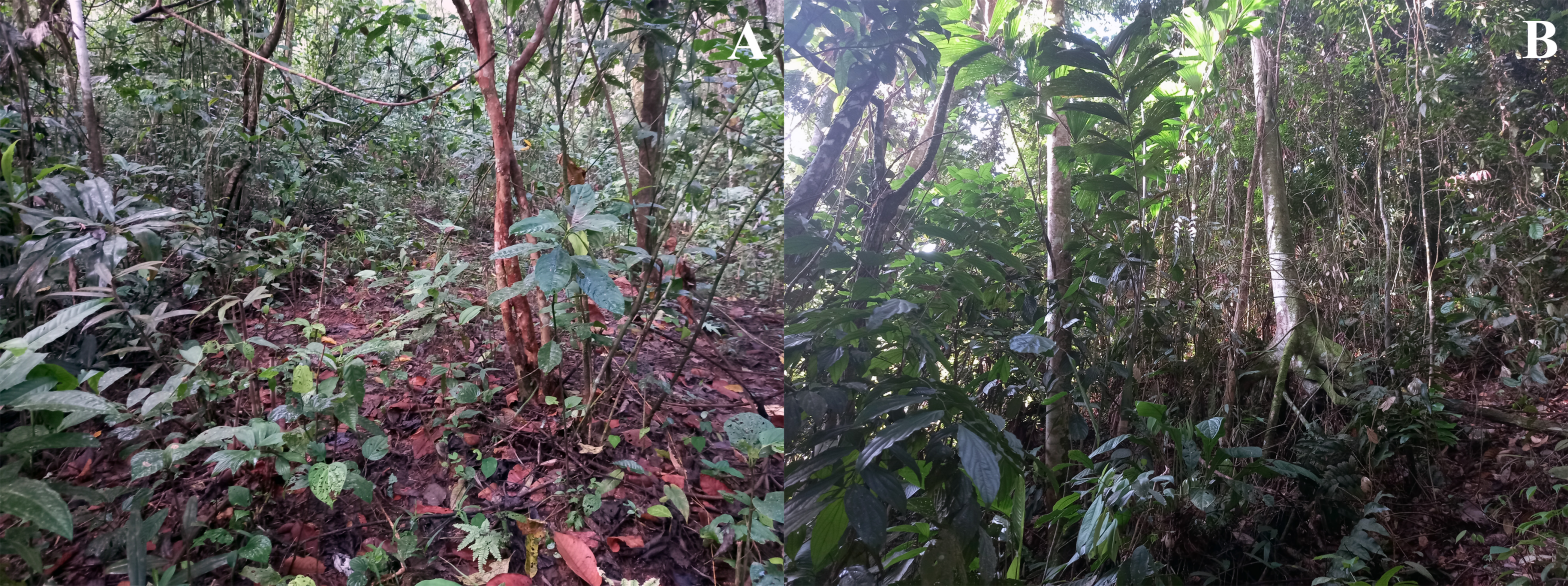
Land cover types across *Ameerega ingeri* localities. (A) Open areas. (B) Non-open areas. Photographs by Yulfreiler Garavito-David.

In the locality of San Jose del Fragua (SJF), we delimited four transects, each measuring 70 × 20 m, across two sites that included both open and non-open areas, each with a relatively homogeneous structure (Fig. 3). These fragments are natural and have not been affected by anthropic activities in the past decade. The classification of open and non-open areas followed a modified version of the methodology used in recent studies (*e.g.*, Ribeiro & Walter, 1998), adapted to the vegetation of the Andean-Amazonian piedmont.

This approach involved grouping habitat characteristics relevant to certain habitats used for some neotropical anurans (Table 1). Collections were made using the time-limited active search method (Crump & Scott Jr, 1994; McDiarmid et al., 2012) between 09:00 to 12:00 h (diurnal) and 19:00 to 21:00 h (nocturnal).

**Table 1:** Criteria used to distinguish open *vs.* non-open areas in the habitat of *Ameerega ingeri*.

Transect	Location		Elevation	Description
	Latitude	Longitude		
Non-open areas	1.354773 N	75.972925 W	560	Closed canopy forest with dense vegetation at the tree level (trees 12–15 m tall), a well-developed shrub layer (<50 cm), and herbaceous cover.
	1.349132 N	75.998065 W	507	
Open areas	1.355260 N	75.971341 W	513	Open or semi-closed canopy with sparse vegetation at the tree level (trees up to 12 m), with scattered shrubs and herbaceous plants.
	1.348559 N	75.997722 W	483	

### Populations and territoriality

**Figure 4:**
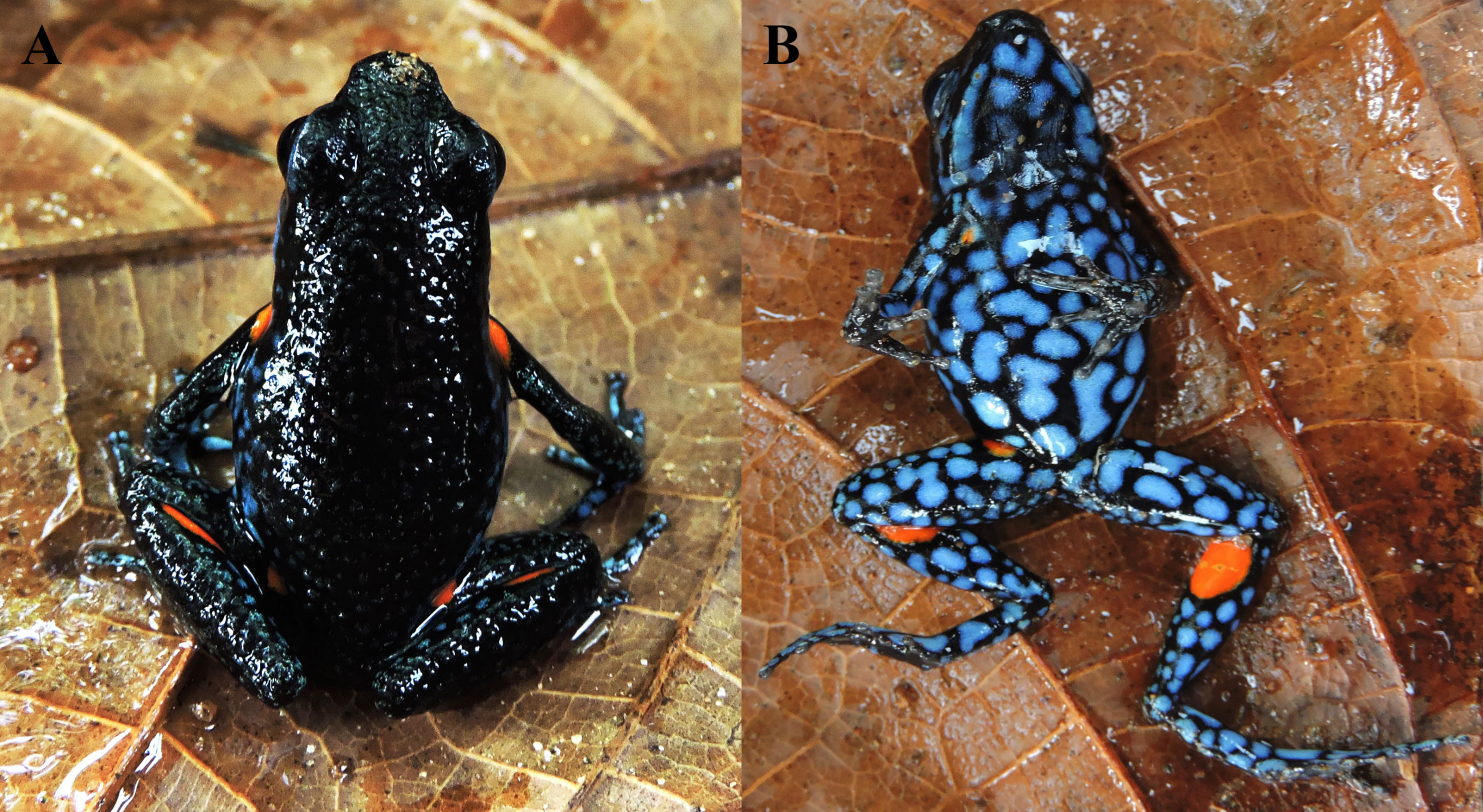
Coloration patterns of *Ameerega ingeri*. (Voucher UAM-H 2054; SVL: 20.40 mm) (A) Dorsal view. (B) Ventral view. Photos by Alejandro Navarro-Morales.

In SJF we recorded *A. ingeri* in sympatry with *A. hahneli* (Cochran & Goin, 1970). These species show high morphological similarity, but they are diagnosed from each other based on coloration and external morphological patterns (Boulenger, 1884; Cochran & Goin, 1970; Twomey & Brown, 2008). To identify individuals and detect potential recaptures of *A. ingeri*, we photographed the ventral and dorsolateral coloration of each specimen (Fig. 4). This non-invasive method has been successfully applied to other anuran species (*e.g.*, Caorsi, Santos & Grant, 2012; Basto-Riascos, López-Caro & Vargas-Salinas, 2017; Diaz-Ricaurte, Guevara-Molina & Serrano, 2019; Fiorillo et al., 2023). To assess individual recaptures, we initially performed manual visual comparisons between photographs. Additionally, to ensure that the observed dorsal spot patterns were not repeated among individuals, we employed computer-assisted matching (CAM) using the software Wild-ID (Wildlife Pattern Recognition Software, v.1.0, Hanover, NH, USA; Bolger et al., 2011). Besides, to determine the possible territoriality, each individual was assigned a registration number, as well as the coordinate of the capture location.

### Natural history and habitat use

We recorded various aspects of natural history, including reproductive biology, vocalization preferences, and habitat use. This involved documenting the specific location within the transect where each individual was found (either in open or non-open areas), the activity it was engaged in (*e.g.*, inactive, vocalizing, foraging, in amplexus, transporting tadpoles), and the type of substrate on which the activity was occurring (*e.g.*, green leaves, ground, leaf litter; *e.g.*, Basto-Riascos, López-Caro & Vargas-Salinas, 2017; Diaz-Ricaurte, Guevara-Molina & Serrano, 2019).

### Analysis of stomach contents

We examined the stomachs of nine specimens through a ventral longitudinal incision. Of these, only four specimens contained identifiable prey items. Stomach contents were examined under a LEICA EZ4 stereomicroscope, and prey items were identified to the lowest possible taxonomic level (*e.g.*, Magnusson et al., 2003; Solé & Rödder, 2010).

For each specimen, we recorded the abundance (N) of each prey category by counting the number of individual prey items. The biomass of stomach contents (PI) was measured using an analytical balance (±0.0001 g). Average values for abundance and biomass were calculated across all four specimens containing prey. We calculated the frequency of occurrence (%FO) for each prey category using the formula: %FO = (n/N) * 100, where n is the number of stomachs in which a given prey category occurred, and N is the total number of stomachs with contents. This metric reflects how commonly each prey type was consumed (Starck II & Schroeder, 1971). To assess the relative importance of each prey category, we calculated the Importance Index (I) as: I = (%FO + %N + %PI)/3, following Biavati, Wiederhecher & Colli (2004), with modifications from Méndez (2020), where: %N = relative numerical abundance of a prey type, and %PI = relative biomass proportion of that prey type.

We also estimated the trophic niche breadth of the population using Levin’s index: *B* = 1/Σ(Pi^2^), where *Pi* is the proportional abundance of prey category *i*. The standardized niche breadth (BA) was then calculated using: BA = (B − 1)/(n − 1), where *n* is the total number of prey categories, and BA ranges from 0 (specialist) to 1 (generalist) (Krebs, 1989).

### Advertisement call

We recorded six calls from two males (850 analyzed notes) with a DR-40 WL digital recorder (Tascam, Montebello, CA, USA) at a sampling rate of 44.1 kHz and a sample size of 16 bits. Recordings were made on June 26, 2024, between 05:40 and 06:30 h, with temperatures ranging from 23 to 25 °C. We analyzed calls using Raven Pro 1.6, 64-bit version (Bioacoustics Research Program, 2014) with the following settings: window type = Hann, window size = 256 samples, 3 dB filter bandwidth = 270 Hz, brightness = 50%, contrast = 50%, overlap = 85% (locked), color map = “Cool”, DFT size = 1024 samples (locked), grid spacing (spectral resolution) =46.9 Hz. We analyzed temporal traits in oscillograms and spectral traits in spectrograms (Köhler et al., 2017).

We used the ‘Peak Frequency’ function to determine the peak of dominant frequency; frequency values with 5% and 95% of call energy were obtained by “Frequency 5% (Hz)” and “Frequency 95% (Hz)” functions and were considered as the minimum and maximum dominant frequencies (Hz), respectively (Charif, Waack & Strickman, 2010). Frequency modulation was accessed through the “1st Quartile Frequency” and “3rd Quartile Frequency” functions; these Raven functions provide the frequencies that break the selection into two frequency intervals containing 25 and 75% of the energy in the selection, respectively (Charif, Waack & Strickman, 2010).

We used the *seewave* v. 2.1.8 package (Sueur, Aubin & Simonis, 2008), R platform (version 4.1.1 “Kick Things”; R Core Team, 2021) to generate figures. Seewave settings for the spectrograms were: Hanning window, 85% overlap, and 512 points resolution (FFT). Sound files analyzed are TASCAM_0884 *A ingeri*-1 individuo_edited, TASCAM_0885 *A ingeri*-2 individuo_edited. Sound files analyzed were deposited in the Fonoteca Neotropical Jacques Vielliard (FNJV; labels FNJV 123004–123005), Museu de Diversidade Biológica (MDBio), Instituto de Biologia, Universidade Estadual de Campinas (UNICAMP).

We calculated means and standard deviations of the species using the average values of individual males, while the ranges (variation) represent the minimum and the maximum values observed across all call samples (raw data). Note and call terminologies follow Köhler et al. (2017). To evaluate between-male call variation, we calculated the coefficients of variation (CV = [standard deviation/mean] ×100). As outlined by Gerhardt (1991), static call properties generally exhibit low between-male variability (CV < 11%), whereas dynamic properties are characterized by higher variability (CV > 15%).

### Estimation of Extent of Occurrence, Area of Habitat, and Area of Occupancy

We carry out a search for *A. ingeri* distribution records from literature and databases of Natural History Museums in Colombia (Appendix I). These records were used to map the species” Extent of Occurrence (EOO), Area of Occupancy (AOO) and Area of Habitat (AOH). According to Lumbierres et al. (2022) these range-related metrics represent crucial sources of information for effective conservation, especially for species classified in some categories of extinction risk by The International Union for Conservation of Nature’s Red List of Threatened Species (IUCN Red List) (IUCN, 2012; Brooks et al., 2019; IUCN, 2019).

The EOO represents the total spatial area encompassing a species’ known occurrences, and indicates its potential extinction risk. When combined with the AOO, it allows to determine the distribution areas essential for the species’ survival, based on specific geographical records and a 4 km^2^ grid cell size (IUCN, 2019; Kass et al., 2021; Serrano et al., 2020; Serrano et al., 2023). However, The AOH allows the identification of suitable habitat for the species, considering factors such as land cover type and elevation range (Brooks et al., 2019; Lumbierres et al., 2022).

We calculated the EOO and AOO using GeoCAT (Bachman et al., 2011) following IUCN assessment criteria (Brooks et al., 2019). We also calculate the AOH using Google Earth Engine and MapBiomas Amazonia initiative for the year of 2023 (Gorelick et al., 2017; MapBiomas, 2024) to determine the total area occupied by *A. ingeri* in Gallery and Secondary Forest within Moist Forest and Andean-Amazonian ecoregions, at elevations between 214 to 1,285 m asl. We used QGIS 3.24 (QGIS Development Team, 2022) to map the EOO and AOO for *A. ingeri*. By combining these complementary geographic range measures, we can obtain precise information on species’ distribution and habitat suitability. This knowledge is essential for developing effective conservation strategies.

### Molecular data

Whole genomic DNA was extracted from muscle or liver tissues of five specimens of *Ameerega ingeri* using a Qiagen DNeasy kit (Valencia, CA, USA) following the manufacturer’s protocol. Next, we amplified a fragment of the mitochondrial 16S gene using primers 16Sar and 16Sbr (Palumbi et al., 2002). For the amplification reactions using polymerase chain reaction (PCR), we prepared a mixture containing 7.5 µl of Taq DNA Polymerase Master Mix (Ampliqon S/A, Denmark), 0.4 µl of each primer at 10 µM (forward and reverse), and 1–2 µl of genomic DNA, adjusting the total reaction volume to 15 µl with Milli-Q water. We targeted a fragment of the mitochondrial 16S rRNA gene using the primers 16Sar and 16Sbr described by Palumbi (1996). The PCR cycling conditions included an initial denaturation at 94 °C for 3 min, followed by 35 cycles of denaturation at 94 °C for 20 s, annealing at 50 °C for 20 s, and extension at 68 °C for 40 s, ending with a final extension at 68 °C for 5 min. The resulting PCR products were purified and sequenced by Eurofins Genomics Inc. (Louisville, KY, USA).

We compared the newly obtained 16S sequences with all *Ameerega* sequences available in GenBank that overlapped with the same fragment region, representing 21 recognized species. Sequence alignment of the mitochondrial 16S gene fragments was conducted using the MAFFT algorithm (Katoh et al., 2002) implemented in Geneious v 9.0.5, applying default parameters. The final alignment consisted of 296 sequences spanning a 605 base pair (bp) fragment of the 16S gene. All GenBank accession numbers and voucher specimens included in this study are provided in Table S2.

Phylogenetic relationships were inferred using maximum likelihood (ML) analyses in RAxML (Stamatakis, 2014), executed *via* raxmlGUI 2.0 (Edler et al., 2021). The ML analyses were performed under the JC substitution model with rapid bootstrap, employing 1,000 bootstrap replicates. The choice of substitution model was evaluated using Modeltest (Darriba et al., 2020) within raxmlGUI 2.0.

For species delimitation, we applied the Poisson Tree Processes (PTP) method (Zhang et al., 2013) based on the inferred ML tree. The PTP analyses were conducted through the web server (http://species.h-its.org/ptp/) using 500,000 MCMC generations, with a thinning interval of 100 and a burn-in of 10%. Additionally, we employed the distance-based Assemble Species by Automatic Partitioning (ASAP; Puillandre, Brouillet & Achaz, 2021) method. This analysis was performed on the online ASAP platform (https://bio.tools/asap-assemble) using a simple distance model to calculate pairwise genetic distances, with all other parameters set to default. We retained the partitioning scheme corresponding to the lowest ASAP score, as recommended by Puillandre, Brouillet & Achaz (2021). Finally, we calculated sequence divergences (uncorrected p-distances) among species/individuals using MEGA v 11.0.13 (Kumar, Nagarajan & Uchil, 2018).

## Results

### Species account

*Ameerega ingeri* (Cochran & Goin, 1970).

***Chresonyms.*** *Dendrobates ingeri*: (Cochran & Goin, 1970): 16, and (Myers, Daly & Malkin, 1978): 332; *Phyllobates ingeri*: (Silverstone, 1976): 35; *Epipedobates ingeri*: (Myers, 1987): 303; *Pseudendrobates ingeri*: (Bauer, 1988): 6; *Ameerega ingeri*: (Grant et al., 2006), 164.

**Figure 5:**
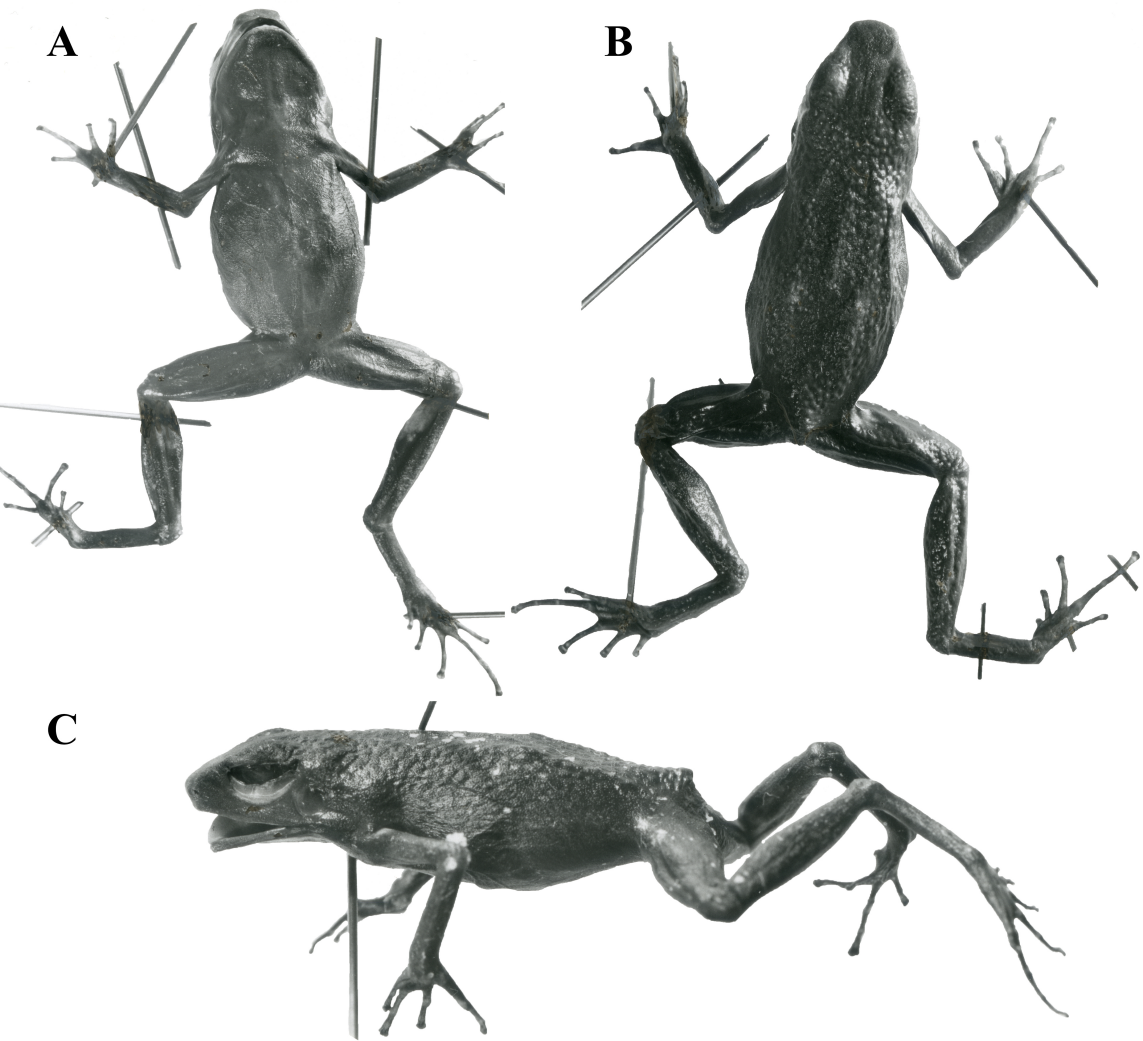
Holotype of *Ameerega ingeri* - USNM 146846. (A) Ventral view. (B) Dorsal view. (C) Lateral view. (Image source: Division of Amphibians & Reptiles Collections of Smithsonian National Museum of Natural History; https://collections.nmnh.si.edu/search/herps/ (http://n2t.net/ark:/65665/34cd60f55-d227-4653-93d5-a30c6f1a259f).

***Holotype*.** United States National Museum (USNM) 146846, Aserrío, near Río Pescado, Caquetá, Combina. Collected by Nicéforo María (Fig. 5).

***Paratypes*.** USNM 146847–9 in the same locality and period of the holotype.

***Type*** ***locality*.** COLOMBIA. Caquetá: Asserío, 20 km from junction Ríos Pescado and Orteguaza, 5 km from right bank Río Pescado (Silverstone, 1976).

***Etymology*.** The specific epithet of this species honors the American zoologist Robert F. Inger, in recognition of his contributions to the ichthyology and herpetology of Borneo. The common name “Niceforo’s Poison Frog”, on the other hand, refers to Brother Nicéforo María, who collected the holotype of the species.

### Diagnosis

*Ameerega ingeri* is assigned to the genus *Ameerega* by the following set of characters: (1) first finger longer than second; (2) webbing absent between the toes, (3) dorsal skin granular (Myers, 1982; Grant et al., 2006). *A. ingeri* is characterized by the following combination of characters: (1) adult mean SVL 21.5 ± 2.4 mm (range = 14.2–24.3) in males and 23.7 ± 1.2 mm (range = 20.4–25.6) in females (Table 2); (2) dorsal skin granular, black, lacking light labial and lateral stripes; (3) orange or yellow spots present above the groin, under axillae, and on shanks (bright spot in the calf region); (4) tadpole with body and tail light tan with grayish brown to dark gray flecks; and (5) advertisement call composed of stereotyped tonal notes (non-pulsed) that are emitted in pairs throughout its duration.

**Table 2:** Morphometry of adult specimens of the new records of *Ameerega ingeri*. (*n*) Number of individuals. mean ± (SD) Standard deviation and range. The abbreviations of measurements are specified in ‘Material and Methods’.

Character	Males (*n* = 21)		Females (*n* = 18)	
	Mean ± SD (mm)	Range (mm)	Mean ± SD (mm)	Range (mm)
SVL	21.5 ± 2.4	14.2–24.3	23.7 ± 1.2	20.4–25.6
FL	10.1 ± 1.2	7.1–12.4	11.0 ± 0.6	9.8–11.6
TL	10.9 ± 1.0	7.9–12.8	11.8 ± 0.6	10.5–12.9
FoL	9.6 ± 1.0	6.6–11.0	10.2 ± 0.5	9.1–11.2
HaL	5.8 ± 0.6	4.0–6.9	6.2 ± 0.3	5.6–6.6
HL	7.3 ± 0.6	5.8–8.4	7.5 ± 0.4	6.4–8.2
HW	7.0 ± 0.8	4.7–8.2	7.4 ± 0.5	6.8–8.2
BW	7.1 ± 1.1	5.0–9.5	7.3 ± 1.0	5.3–8.9
UEW	1.8 ± 0.2	1.3–2.0	1.8 ± 0.2	1.1–2.2
IOD	2.3 ± 0.3	1.5–2.7	2.4 ± 0.2	1.9–2.7
IND	3.2 ± 0.3	2.3–3.7	3.3 ± 0.3	2.6–4.0
TD	1.0 ± 0.1	0.7–1.3	1.1 ± 0.2	0.9–1.4
ED	3.1 ± 0.3	19–3.4	3.3 ± 0.3	3.0–4.0
DET	0.5 ± 0.1	0.3–0.7	0.5 ± 0.1	0.4–0.6
L1F	4.6 ± 0.5	3.1–5.5	5.0 ± 1.2	4.3–5.3
L2F	4.0 ± 0.5	2.8–5.0	4.3 ± 0.2	3.8–4.6
W3D	0.5 ± 0.1	0.3–0.6	0.5 ± 0.1	0.3–0.6
W3F	0.4 ± 0.1	0.2–0.5	0.4 ± 0.1	0.2–0.5

**Figure 6:**
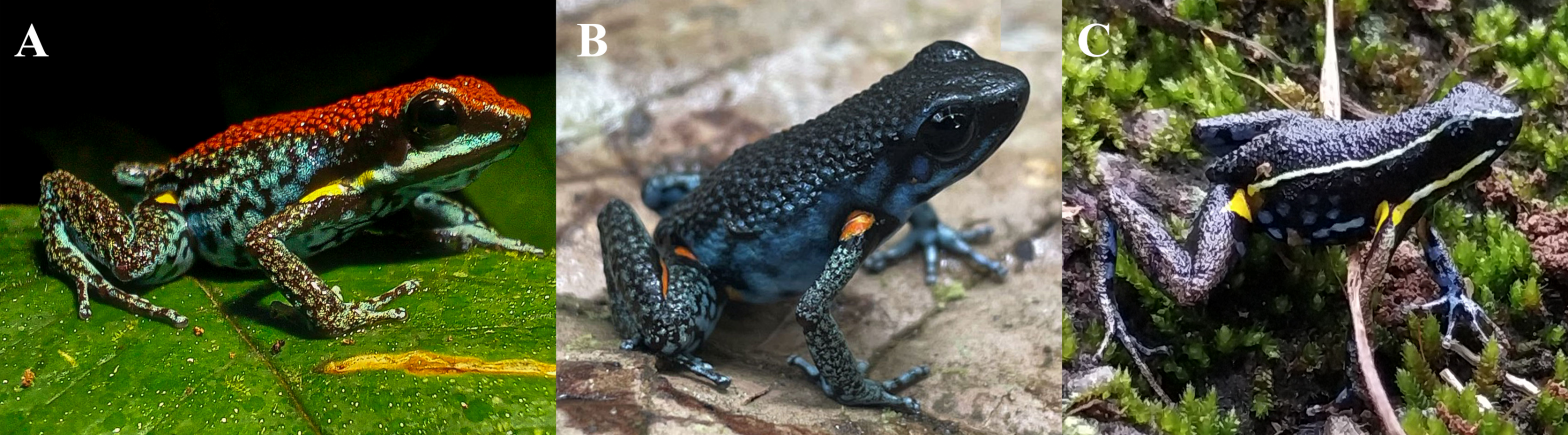
External morphology of *Ameerega ingeri* in comparison with congeneric species. (A) *A. bilinguis*, (B) *A. ingeri*, and (C) *A. hahneli*. Photos by Yulfreiler Garavito-David (A) and Alejandro Navarro-Morales (B, C).

**Figure 7:**
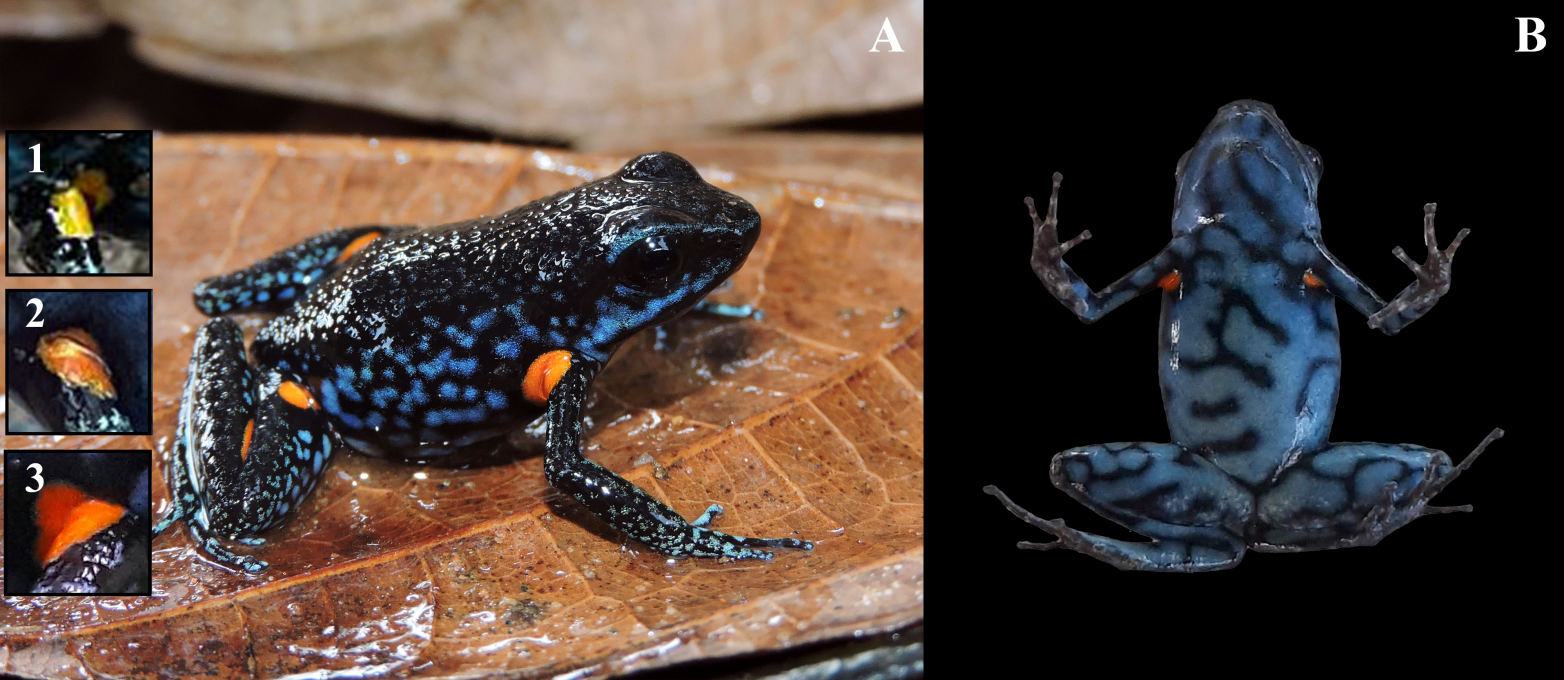
Adult male of *Ameerega ingeri* (UAM-H 2049; SVL = 21.49 mm) of the locality Reserva Natural y Agroturistica Doña Blanca. (A) Lateral view. (B) Ventral view. (Inset 1) Yellow. (Inset 2) Yellow with orange. (Inset 3) Light orange. Photos by Alejandro Navarro-Morales (A) and Yulfreiler Garavito-David (B).

*Ameerega bilinguis*, *A. hahneli*, and *A. parvula* are the Amazonian sympatric species that most closely resemble *A. ingeri*, but they can be readily distinguished based on coloration, body pattern, and acoustic traits. *Ameerega ingeri* differs from *A. bilinguis*, which has a dark red dorsum, a yellow, light blue, or white labial stripe, and lacks spots in the calf region (Fig. 6A) (Jungfer, 1989). In contrast, *A. ingeri* exhibits a dark dorsum with distinctive orange spots in the calf region (Figs. 6B–7). In the context of acoustic comparisons, *A. bilinguis* exhibits the advertisement call most similar to *A. ingeri*, showing overlaps in all the temporal and spectral traits (Jungfer, 1989), except for the interval within note pairs, which refers to the silence between the two notes composing the pair. In *A. bilinguis*, this interval ranged from 68–84 ms (*x* = 78, *n* = 10; Jungfer, 1989), whereas in *A. ingeri*, it ranged from 28–71 ms (*x* = 49). There is no available data for the note emission rate of *A. bilinguis* to compare these two species; however, we think there will not be much difference given the similarity in the other temporal traits of both (Jungfer, 1989).

When compared to *A. hahneli*, *A. ingeri* can also be easily distinguished. *Ameerega hahneli* has a brown dorsum and limbs, with or without black spots, a yellow or white labial and dorsolateral stripe, and yellow oval spots in the concealed areas of the axillae, groin, and calves (Fig. 6C) (Rodríguez & Duellman, 1994). In contrast, *A. ingeri* has a dark dorsum and limbs with small blue spots on the limbs and orange or yellow spots on other body regions (Figs. 6B–7). Finally, *A. ingeri* differs from *A. parvula*, which has a red dorsum with an incomplete light lateral stripe, a teal belly with irregular black bands, and lacks spots in the concealed regions (Silverstone, 1976). In contrast, *A. ingeri* presents a dark dorsum, a light blue belly, and conspicuous spots on the body. Additionally, *A. hahneli* and *A. parvula* have calls composed of single notes, *i.e.,* notes not arranged in pairs, whereas *A. ingeri* and *A. bilinguis* emit calls composed of paired notes. Therefore, advertisement calls easily differentiate *A. ingeri* from *A. hahneli* and *A. parvula* (Duellman, 1978; Haddad & Martins, 1994; Twomey & Brown, 2008; Serrano-Rojas et al., 2017; Brown et al., 2019).

*Ameerega ingeri* differs from other species described such as *A. altamazonica, A. bassleri, A. boliviana, A. cainarachi, A. ignipedis, A. macero, A. munduruku, A. panguana, A. pepperi, A. petersi, A. picta, A. planipaleae, A. pongoensis, A. pulchripecta, A. rubiventris, A. shihuemoy, A. silverstonei, A. simulans, A. smaragdina, A. trivittata*, and *A. yoshina* (Silverstone, 1976; Rodríguez & Myers, 1993; Rodríguez & Duellman, 1994; Morales & Velazco, 1998; Myers, Rodríguez & Icochea, 1998; Lötters et al., 1997; Gonzales, Lötters & Reichle, 1999; Reichle & Aguayo, 2006; Roberts et al., 2006; Lötters et al., 2007; Medina-Muller & Chávez, 2007; Twomey & Brown, 2008; Brown & Twomey, 2009; Neves et al., 2017; Serrano-Rojas et al., 2017; Brown et al., 2019) by the absence of lateral and labial stripes. However, some species such as *A. braccata, A. berohoka, A. flavopicta, and A. silverstonei* also lack body stripes, but differ in dorsal coloration and the presence of spots or stripes on the dorsum. In *A. ingeri*, the dorsum is uniformly dark and lacks any spots, whereas *A. braccata* has a brown dorsum with golden, yellow, or white spots (Haddad & Martins, 1994); *Ameerega berohoka* has irregularly spaced cream spots (Vaz-Silva & Maciel, 2011); *A. flavopicta* bears white or yellow spots or stripes (Lötters et al., 2009); and *A. silverstonei* exhibits a yellow, orange, or red dorsum (Myers & Daly, 1979). Additionally, the venter of *A. ingeri* is blue with black reticulations, in contrast to the light grey to light brown in *A. boehmei* (Lötters et al., 2009), and the white or cream venter of *A. imasmari* (Brown et al., 2019).

As in *A. ingeri*, the notes emitted by *A. petersi* can be arranged in pairs; however, *A. petersi* can also emit individual notes or groups of three or four notes (Schlüter, 1980; Myers, Rodríguez & Icochea, 1998). The pairs of notes are highly stereotyped in *A. ingeri*, with no groups of three or four notes observed, and individual notes are only observed at the beginning of some calls (See the “Advertisement call” section for more details). Additionally, when the notes are arranged in pairs in *A. petersi*, the interval within the pairs is longer (80–110 ms) compared to *A. ingeri* (Schlüter, 1980; Myers, Rodríguez & Icochea, 1998).

Another species that can also emit notes in pairs is *A. pongoensis*, which has a call with irregularly spaced notes, with a note (or pair of notes) observed every two to three seconds (Twomey & Brown, 2008). The note emission rate in *A. ingeri* is higher and varies very little. Furthermore, the advertisement call of *A. ingeri* consists of regularly spaced notes, unlike that of *A. pongoensis*.

This presence of pairs of notes in its call easily distinguishes *A. ingeri* from all other 22 congeneric species with known calls, which all have single notes, *i.e.,* notes not arranged in pairs (Myers & Daly, 1979; Schlüter, 1980; Rodríguez & Myers, 1993; Haddad & Martins, 1994; de La Riva, Márquez & Bosch, 1996; Lötters et al., 1997; Myers, Rodríguez & Icochea, 1998; Lötters, Reichle & Jungfer, 2003; Lötters et al., 2007; Twomey & Brown, 2008; Brown & Twomey, 2009; Lötters et al., 2009; Forti, Strüssmann & Mott, 2010; Magrini et al., 2010; Martins & Giaretta, 2012; Andrade et al., 2014; Costa-Campos, Lima & Amézquita, 2016; Neves et al., 2017; Serrano-Rojas et al., 2017; Brown et al., 2019). There is no available acoustic data for *A. planipaleae*, so we did not include it in the comparison.

### Color in life

Dorsal skin is granular with a dark pigmentation. The dorsolateral region exhibits a dark base color with irregular dark blue spots extending from the cephalic to the inguinal region. Some specimens display a dark blue stripe from the nostril to the anterior part of the tympanum, bordering the upper eyelid. Limbs have a dark base color on the dorsal surface with small, irregular, dark blue spots that become blurred toward the phalanges, transitioning into a light blue base color with irregular black spots. The pupil is dark. Specimens exhibit orange or yellow oval spots in concealed axillary and calf regions (Fig. 7A). The belly displays a pattern of irregular light blue spots bordered by black coloration of varying intensity. In some individuals, the buccal and pectoral regions are slightly darker than other belly areas (Fig. 7B). Metamorphosing individuals exhibit yellow spots, while some subadults display orange spots. Adults typically have bright orange spots. Additionally, certain individuals may have a bright blue canthal stripe and *canthus rostralis* (Fig. 7).

### External adult morphology

Medium-sized species; males up to 24.3 mm, females up to 25.6 mm in snout–vent length (SVL) (Table 2). Head widest between jaw articulations and tympana; narrower than body; maximum head width 31.72% of SVL. Eyes very protuberant. Tongue oval, medium-sized. Premaxillary and maxillary teeth present. Vocal slits present. Dorsal skin of head coarsely granular; skin on limbs smooth or nearly smooth; sides of head and body, and ventral surfaces smooth. For detailed voucher information, see Table S3.

The snout presents a distinctive profile: sloping laterally, bluntly rounded dorsally, and truncate ventrally. Its nares are oriented posterolaterally from the tip, discernible from both frontal and ventral views, yet concealed from above. The *canthus rostralis* is sloped and gently rounded, leading to a loreal region that is nearly vertical and subtly concave. Notably, the interorbital distance is 2.5 times the width of the upper eyelid. The eye is prominent and large, with a maximum diameter equivalent to 14.3% of the snout-vent length (SVL). Its pupil is both round and horizontally elliptical. The tympanum is circular, lacking a tympanic annulus, and its diameter is less than 32.0% of the eye’s diameter (ED). A supratympanic fold is absent.

**Figure 8:**
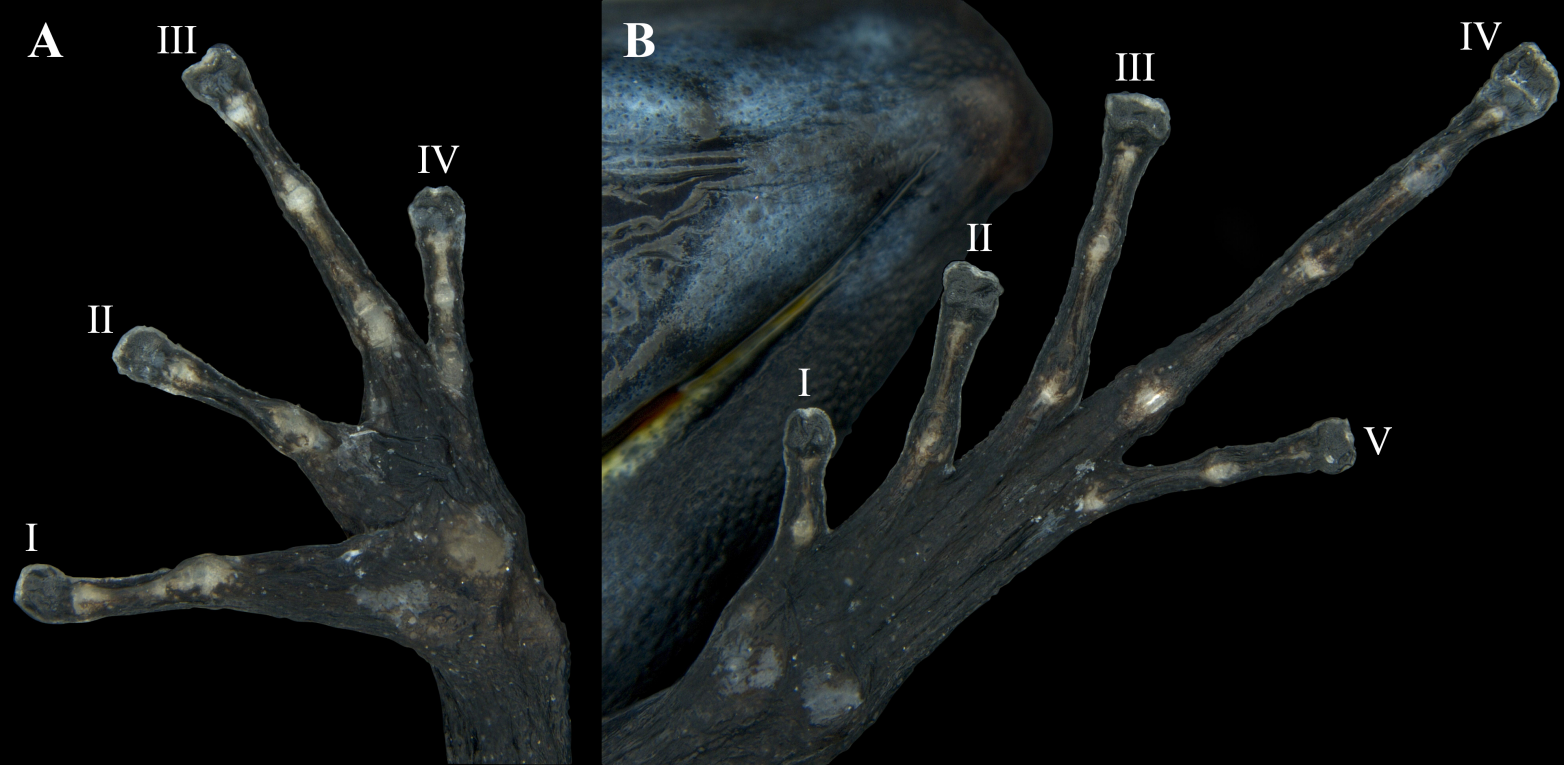
Ventral view of the hand and foot of an adult female *Ameerega ingeri* (voucher UAM-H 1652; SVL = 23.43 mm). (A) Hand; (B) foot. Photos by Alejandro Navarro-Morales.

Hands relatively small, length being 27.0% of SVL. Relative length of addressed fingers II < I < IV < III. Discs weakly to moderately expanded on all fingers. In adults, disc on finger III is 1.25 times wider than the distal end of adjacent phalanx. A large, circular outer metacarpal tubercle present at base of palm and a smaller inner metacarpal tubercle on base of first finger, these relatively low, with rounded surfaces; one subarticular tubercle on fingers I and II, two on fingers III and IV; all subarticular tubercles well developed and raised. No supernumerary tubercles. Keel along the sides of fingers is extremely faint, not forming a fringe (Fig. 8A).

Hind limbs long, with heel of adpressed limb reaching to eye or between eye and tip of snout. Tibia length 44.0% of SVL. Relative lengths of adpressed toes I < II < V < III < IV; No basal webbing or fringes but toes with lateral keels; toes I, II barely expanded (much smaller than finger discs), and toe III, IV, and V expanded (disc 1.5 times broader than adjacent phalanx). One to three moderately elevated subarticular tubercles: one on toes I and II, two on toes III and V, and three on toe IV. Two metatarsal tubercles with low, rounded profiles; inner metatarsal tubercle slightly larger than outer. Supernumerary tubercles absent. Distal half of tarsus with tarsal keel bearing a slightly raised tubercle at proximal end; ventrolateral side of tarsus relatively smooth, not especially rugose or tubercular (Fig. 8B).

### Color in preservative

**Figure 9:**
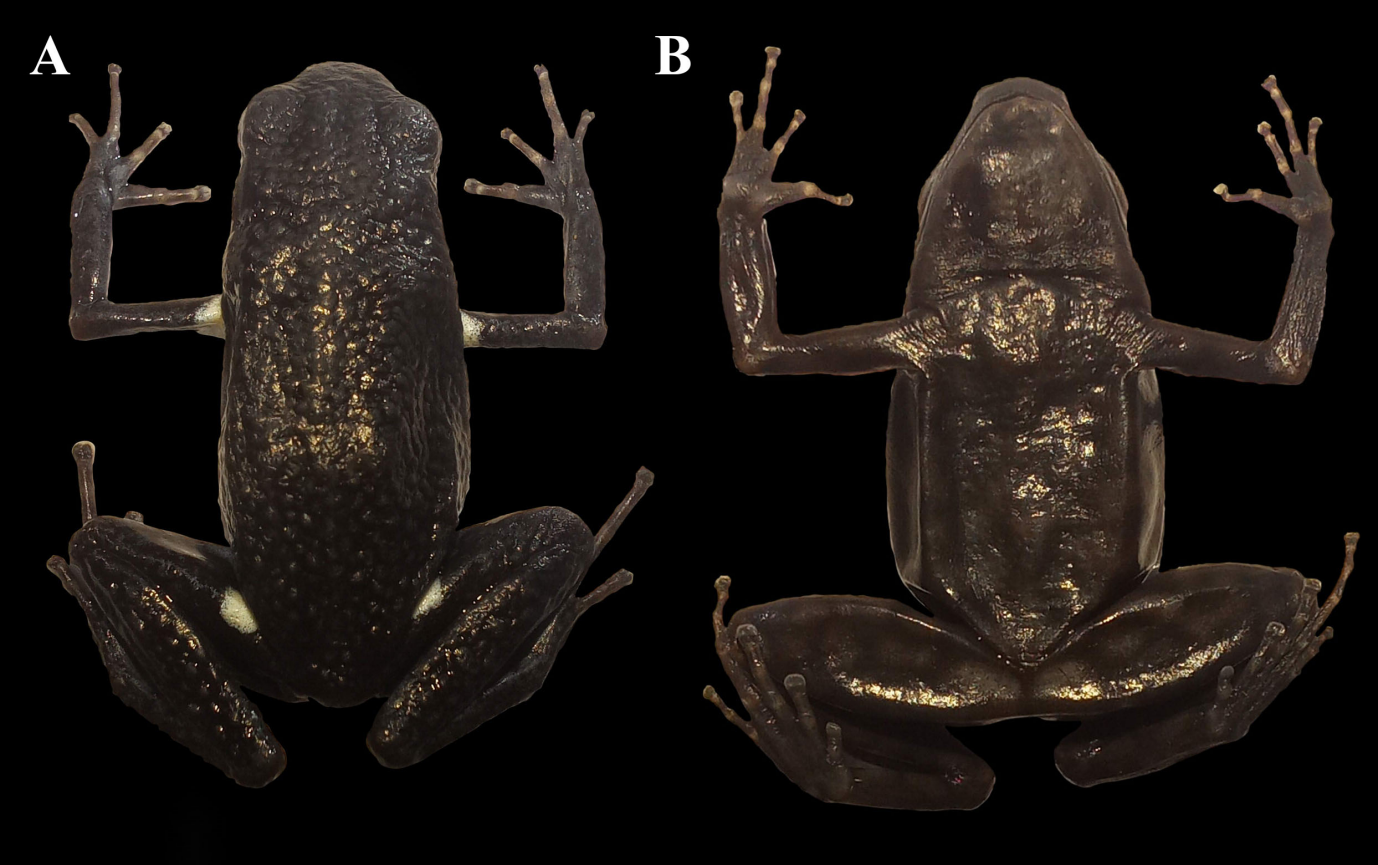
Adult female of *Ameerega ingeri* preserved (Voucher UAM-H 1652; SVL = 23.43 mm). (A) Dorsal view. (B) Ventral view. Photos by Alejandro Navarro-Morales.

The dorsal surfaces of the skull, back, and limbs exhibit a granular texture and dark coloration. Digits have a cream-colored upper part, while some specimens display cream-colored spots in the axillary, inguinal, and calf regions (Fig. 9A). Ventrally, the lower jaw and pectoral region are dark, whereas the belly and femur show weakly pronounced brown reticulations and spots. Some individuals possess two pale white spots in the cloacal region (Fig. 9B).

### Description of Tadpole

The description is based on six tadpoles at stage 35–39 (*sensu* Gosner, 1960). Specimen UAM-H 2067, selected from a sample of 30 larvae collected in a roadside puddle within the Parque Natural Municipal La Resaca, municipality of Belén de Los Andaquíes, Caquetá. Tadpoles had a mean total length of 23.93 mm (min: 22.2–max: 25.99; Standard Deviation, SD: 1.46 mm) and a mean body length of 9.30 mm (8.6–9.8; SD: 0.40 mm). The depressed body had a mean maximum depth of 3.97 mm (3.5–4.7; SD: 0.44 mm) and a mean maximum width of 5.70 mm (5.4–6.1; SD: 0.23 mm). The mean snout was narrowly rounded in the dorsal profile and broadly rounded in the lateral profile (Table 3). A lateral-line system was not visible. The mean tiny nares were indistinct and opened anterodorsally at a mean distance of 0.57 mm (0.3–0.8; SD: 0.19 mm) from the mean snout tip. The mean internarial distance was 1.87 mm (1.7–2.0; SD: 0.12 mm), and the mean interorbital distance was 2.80 mm (2.5–3.1; SD: 0.25 mm). The mean eyes were directed dorsolaterally and had a mean diameter of 1.29 mm. The mean distance from the nostril to the anterior edge of the eye was 0.83 mm (0.6–1.0; SD: 0.15 mm).

**Table 3:** Measurements (in mm) of the tadpoles of *Ameerega ingeri* in the stages 25, 28, 39, 40, 41 *Sensu*Gosner, 1960. (*n*) Number of individuals. mean ± (SD) Standard deviation and range. The abbreviations of measurements are specified in ‘Material and Methods’.

Character	Stage 25 *n* = 5	Stage 26 *n* = 1	Stage 27 *n* = 1	Stage 28 *n* = 2	Stage 29 *n* = 1	Stage 31 *n* = 1	Stage 34 *n* = 1	Stage 35 *n* = 1	Stage 36 *n* = 1	Stage 38 *n* = 1	Stage 39 *n* = 3	Stage 40 *n* = 3	Stage 41 *n* = 7
TL	19.7 ± 2.2 17.2–21.5	–	20.6	18.2 ± 1.4 17.2–19.2	–	–	20.0	25.1	23.9	24.1	23.5 ± 2.1 22.2 –25.9	24.8 ± 0.4 24.4–25.2	25.1 ± 2.1 23.1–29.4
BL	8.1 ± 1.2 6.4–9.3	9.0	8.6	8.1 ± 1.6 6.9–9.2	8.6	9.4	8.8	9.4	9.2	9.3	9.2 ± 0.6 8.6–9.8	9.2 ± 0.6 8.6–9.8	9.1 ± 0.3 8.8–9.6
BH	4.0 ± 0.6 3.5–4.9	3.8	4.4	3.4 ± 0.9 2.7–4.0	3.7	4.1	4.2	4.7	3.7	3.7	3.9 ± 0.4 3.5–4.2	4.2 ± 0.6 3.9–4.9	4.0 ± 0.4 3.7–4.9
BW	4.8 ± 0.4 4.4–5.3	5.4	5.1	4.8 ± 0.8 4.2–5.3	5.1	5.2	5.4	6.1	5.7	5.6	5.6 ± 0.2 5.4–5.7	5.4 ± 0.3 5.2–5.8	5.6 ± 0.2 5.4–5.7
HW	4.1 ± 0.4 3.5–4.4	4.4	4.5	3.9 ± 0.5 3.6–4.3	4.4	4.2	4.2	4.9	4.5	4.5	4.7 ± 0.4 4.4–5.1	4.4 ± 0.1 4.4–4.5	4.5 ± 0.2 4.4–4.7
ED	1.0 ± 0.1 0.9–1.2	1.0	1.3	0.9 ± 0.2 0.8–1.1	1.1	1.1	1.1	1.1	1.3	1.0	1.1 ± 0.1 1.0–1.3	1.1 ± 0.1 1.0–1.1	0.9 ± 0.3 0.4–1.0
END	0.6 ± 0.2 0.4–1.0	1.0	0.8	0.4 ± 0.1 0.3–0.5	0.8	0.4	0.8	0.8	0.8	1.0	0.8 ± 0.1 0.8–1.0	0.8 ± 0.1 0.8–0.9	1.1 ± 0.3 0.8–1.5
NSD	0.3 ± 0.1 0.2–0.5	0.5	0.5	0.2 ± 0.0 0.2–0.2	0.5	0.2	0.6	0.5	0.3	0.8	0.6 ± 0.1 0.5–0.8	0.4 ± 0.4 0.1–0.8	0.4 ± 0.2 0.1–0.7
IND	1.4 ± 0.3 1.1–1.8	1.7	1.6	1.3 ± 0.1 1.2–1.3	1.8	1.3	1.6	2.0	1.8	2.0	1.8 ± 0.1 1.7–1.8	1.7 ± 0.3 1.5–2.0	1.8 ± 0.2 1.5–2.0
IOD	2.0 ± 0.3 1.5–2.2	2.1	2.7	1.8 ± 0.4 1.5–2.1	2.7	2.2	2.7	3.1	3.1	2.8	2.6 ± 0.1 2.5–2.7	2.8 ± 0.2 2.5–2.9	2.8 ± 0.4 2.1–3.1
TAL	11.8 ± 0.9 10.8–12.3		12.1	10.1 ± 0.2 10.0–10.3			11.2	15.6	14.8	14.9	14.3 ± 1.6 13.2–16.1	15.6 ± 0.4 15.1–15.9	15.9 ± 2.3 13.6–20.6
MTH	3.9 ± 0.3 3.5–4.2	4.7	4.4	4.0 ± 1.3 3.1–4.9	4.7	4.2	4.4	4.6	4.4	4.3	4.2 ± 0.2 4.1–4.4	4.3 ± 0.3 4.0–4.5	4.1 ± 0.4 4.0–4.7
TMH	2.1 ± 0.1 2.0–2.2	2.4	2.5	2.3 ± 0.6 1.8–2.7	2.5	2.3	2.0	2.5	2.2	2.0	2.1 ± 0.1 2.0–2.2	2.0 ± 0.5 1.5–2.5	2.1 ± 0.2 1.8–2.5
TMW	2.1 ± 0.5 1.6–2.8	2.1	2.1	1.8 ± 0.6 1.3–2.2	1.8	2.2	2.0	2.1	2.2	2.6	2.3 ± 0.2 2.1–2.5	2.3 ± 0.1 2.2–2.5	2.1 ± 0.1 2.0–2.2
ODW	2.0 ± 0.2 1.7–2.1	2.0	2.1	2.0 ± 0.2 1.8–2.2	2.1	2.2	1.7	2.3	2.2	2.2	2.3 ± 0.2 2.0–2.4	2.0 ± 0.1 1.8–2.1	2.2 ± 0.2 1.8–2.4

**Figure 10:**
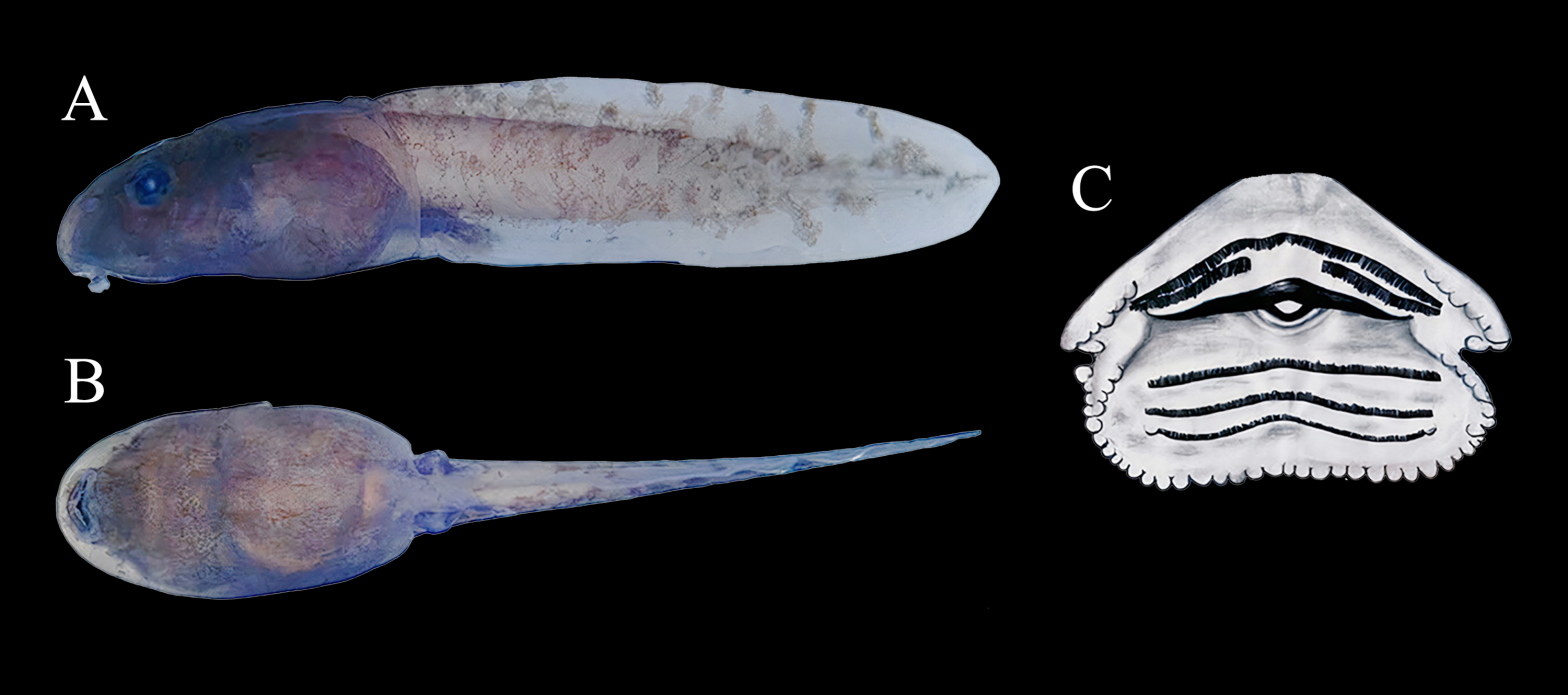
Tadpole of *Ameerega ingeri*. (A) Lateral. (B) Ventral (scale two mm). (C) Oral disc (Gosner stage 36, UAM-H 2067a; scale one mm). Photos by Cesar Malambo.

The spiracle is sinistral, directed posterodorsally as a short, free tube. It opens 4.55 mm from the snout, just below the midline, at 58.9% of the body length. The spiracle tube is 2.6 mm long, with a transverse width of 1.4 mm and an opening diameter of 0.8 mm. The vent tube opening is dextral, 1.46 mm in diameter, and free posteriorly. The tail length is 14.70 mm (13.2–16.1; SD: 1.12 mm). The mean tail muscle height at base of tail was 2.17 mm (2.0–2.5; SD: 0.19 mm), while tail muscle width at base of tail was 2.23 mm (2.10–2.60; SD: 0.20 mm). The oral disc’s ventral width is 38.39% of the maximum body width (Fig. 10). Papillae are absent on the upper lip, which is strongly folded over the A-2 tooth row. The upper jaw sheath is nearly straight, while the lower jaw sheath is V-shaped. Both upper and lower sheaths have fine serrations along their entire length, including the lateral processes. The labial tooth row formula is 2(2)/3. The A-1 tooth row is complete, while A-2 consists of two separated rows with a medial gap. The P-1 tooth row is slightly shorter than P-2, and P-3 is 80% the length of P-1. All P tooth rows are complete (Table 3).

### Color in life of Tadpole

The body is dark brown, densely spotted with darker brown to almost black pigmentation. The tail musculature is pale brown at the junction, gradually fading to transparent posteriorly. The venter is transparent but exhibits slight posterior pigmentation, with a dark brown anterior region and visible intestines through the skin. Hind legs are light tan with pale brown flecks. The tail musculature is speckled with numerous small to moderate-sized, irregular gray to dark brown flecks. The tail is transparent, marked with many irregular, small, dark brown flecks.

### Color in preservation of Tadpole

The body and tail are light tan with grayish-brown to dark gray flecks. The body is almost uniformly heavily pigmented with dark brown. The posterior half of the ventral body is moderately pigmented, while the anterior half is transparent with fewer pale and dark gray spots. The gut is visible. Developing legs are dark brown (Figs. 10A and 10B).

### Ontogenetic variation of Tadpole

Variation in 15 tadpole characteristics across stages 25–41 (*sensu* Gosner, 1960) is presented in Table 2. Total lengths of *A. ingeri* tadpoles ranged from 17.19 to 29.44 mm. The oral disc remained ventrally directed throughout all stages. Papillae were absent on the upper lip across approximately the entire length of the A-1 tooth row. The upper jaw sheath was either straight or slightly V-shaped in all stages, while the lower jaw sheath was consistently V-shaped. The labial tooth row formula was 2(2)/3 for all stages. The A-1 tooth row was complete in all stages. The P-1 tooth row was either as long as or slightly shorter than P-2, and P-3 was always shorter than P-1. The metamorph’s dorsal body color matched that of the adult, but the bright spots on the groin, under the axillae, and on the shanks were yellow.

### Distribution, EOO, AOO and AOH

According to the records reported in this study *A. ingeri* is endemic to Colombia (Figs. 1A and 1B). In Colombia it was recorded in three amazon departments. In Caquetá is distributed in the municipalities of San José del Fragua (Finca El Chiringal, Finca Don Neder, Vereda Cafetales), Belén de los Andaquíes (Finca El Diviso, Finca La Esperanza, La profunda, Las Verdes, Parque Municipal Natural La Resaca, Parque Nacional Natural Alto Fragua-Indi Wasi, Resguardo Indígena La Cerinda, Vereda Aletones, Vereda Tendidos), Florencia (Finca El Ceilan, Finca El Tigre, Finca Sucre, Reserva Natural y Ecoturística La Avispa), El Paujil (Reserva Natural y Agroturística Doña Blanca), Milán (Puerto Diago), Valparaíso (Rio Pescado/Type locality). In the department of Cauca, municipality of Piamonte (Vereda Bajo Congor) and department of Putumayo in two municipalities Mocoa (Vereda La Umbria, Vereda Puerto Limon) and Orito (Vereda El Líbano) (Figs. 1A and 1B; Appendix I).

Consequently, the species’ elevational range extends from 214 to 1285 m asl, based on its known occurrence in more than ten localities, including the seven localities sampled in this study (Fig. 1; Table S1). The species has an Area of Occupancy (AOO) of 92 Km^2^ and an Extent of Occurrence (EOO) of 7,798.333 Km^2^. However, considering the species’ distribution and the habitats it occupies (Forest formations and Flooded forests), the Area of Habitat (AOH) was estimated to be 2,541.961 Km^2^. Additionally, we report new records that extend the known distribution of *A. ingeri*. These new records extend the species’ range approximately 57.02 km from the type locality in Colombia (For detailed information about other localities, please see Table S4).

### Population size, habitat use and territoriality

A total of 94 adult individuals of *A. ingeri* were captured in San José del Fragua between April 2021 to March 2022. However, 32 individuals were recorded in Bélen de los Andaquíes. Photographic records of dorsal and ventral patterns allowed for 100% accuracy in the identification of captured and recaptured individuals. A single recapture was recorded, representing 0.83% of the total number of individuals registered.

**Figure 11:**
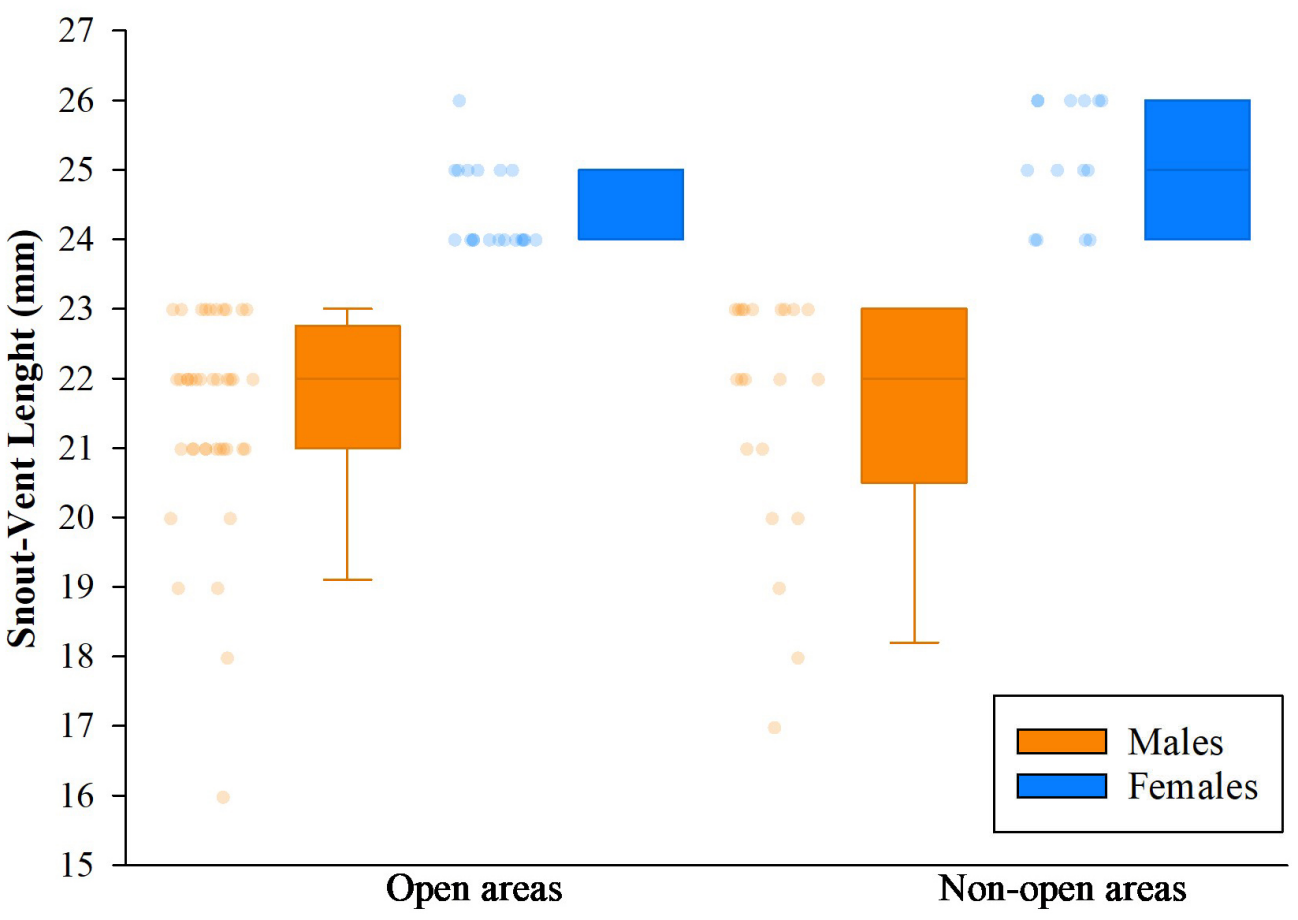
Comparison of body size (SVL) of adults found in San Jose del Fragua. Body size (SVL) of 96 adults found in open and non-open areas in San José del Fragua.

Analysis of variance (ANOVA) and Tukey’s test revealed no significant effect of the delimited sampling areas (open and non-open) on the presence of males or females. However, there were differences in body size, with females larger than males, both in non-open areas (*df* = 33; *n* = 35; *F* = 47.23; *p* < 0.05) like open areas (*df* = 57; *n* = 59; *F* = 67,36; *p* < 0.05). The body size of males between coverage did not present differences (*df* = 59; *n* = 61; *F* = 0.05; *p* > 0.05), but the females did present differences, the largest ones being those found in no-open coverage (*df* = 31; *n* = 33; *F* = 7.96; *p* < 0.05) (Fig. 11).

**Figure 12:**
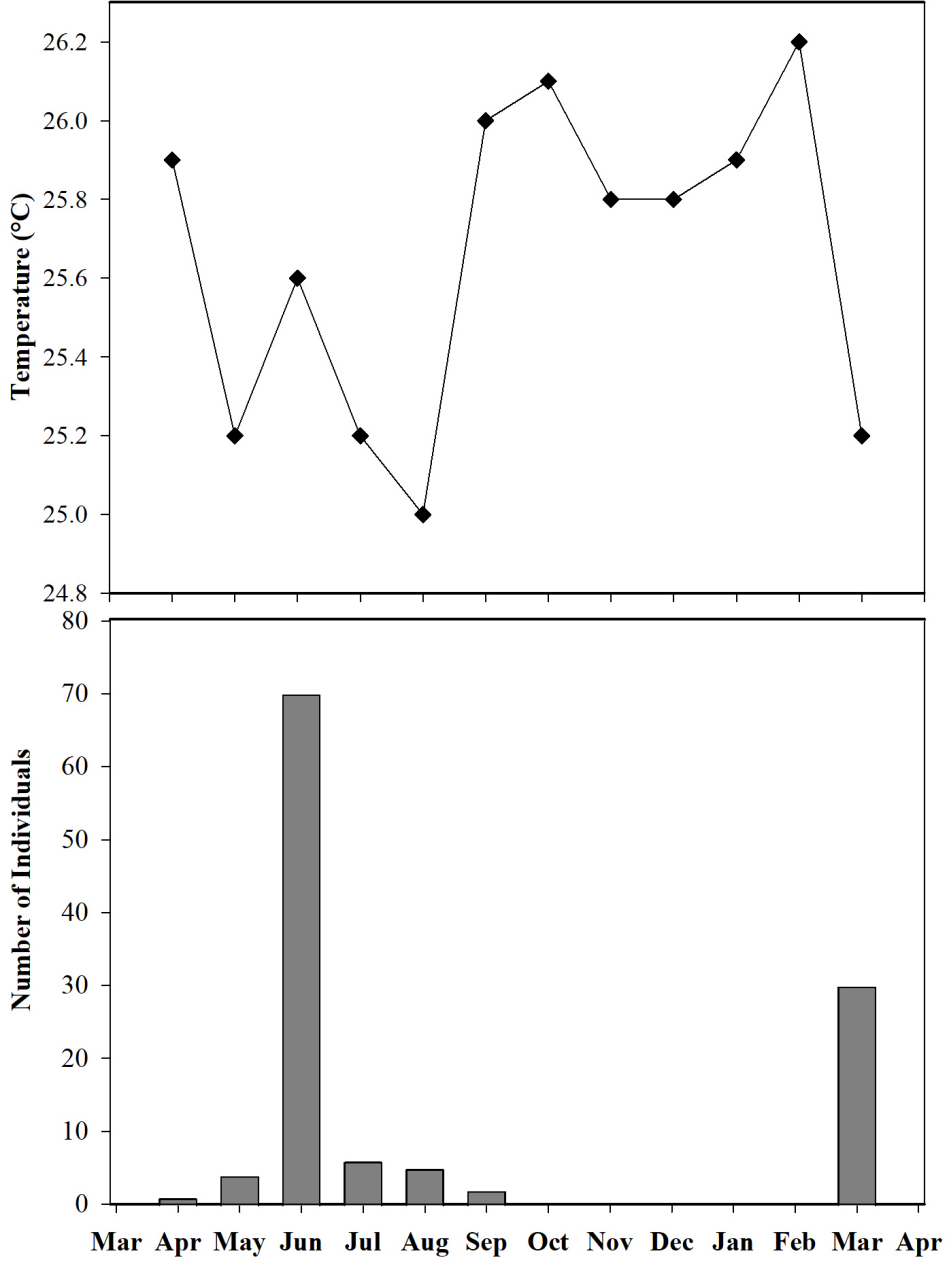
Number of individuals of *Ameerega ingeri* captured during the sampling months *vs* media temperature for each month.

**Figure 13:**
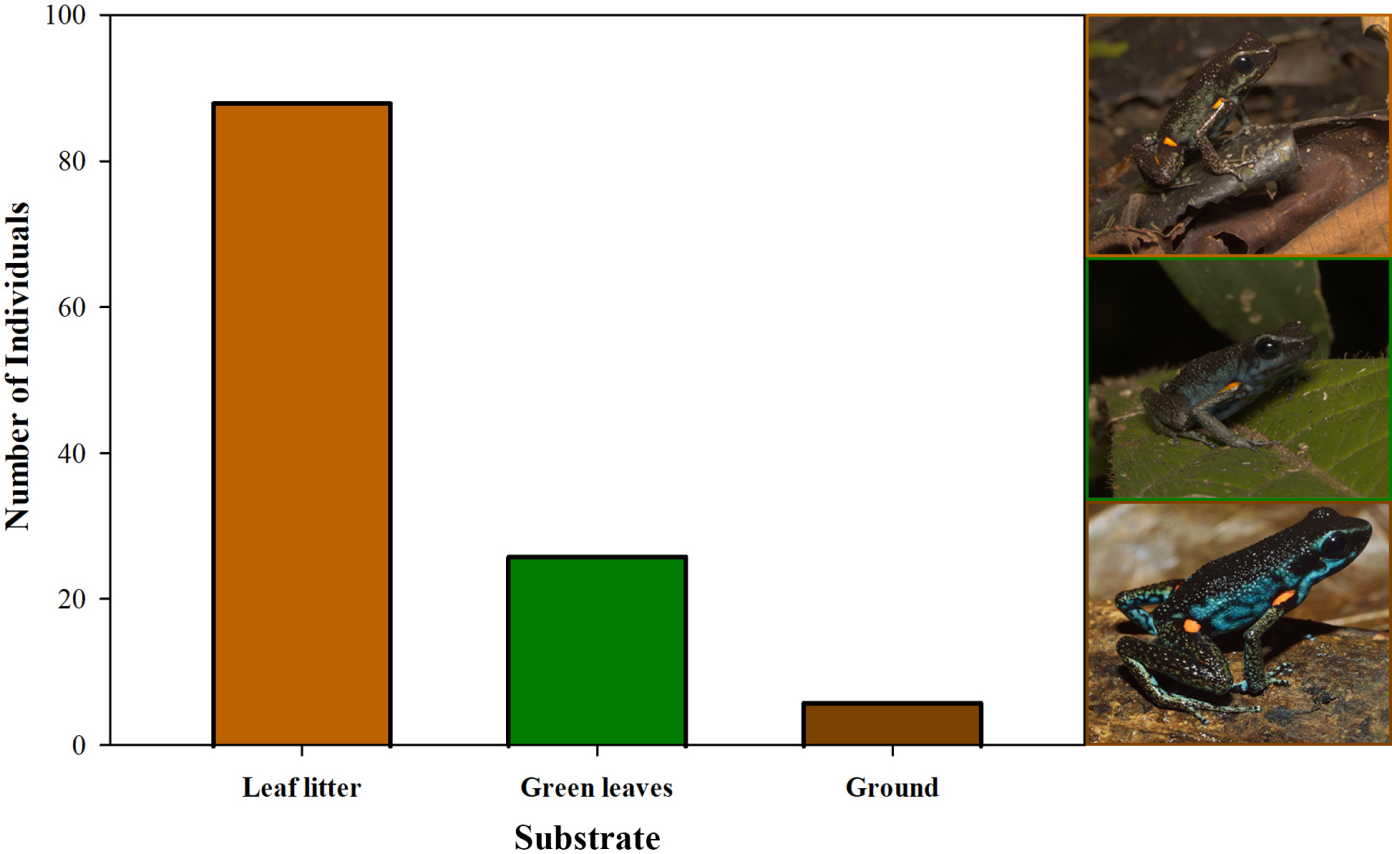
Substrate use by *Ameerega ingeri*. Number of *Ameerega ingeri* individuals recorded on each type of substrate.

Furthermore, we observed that temperature had no influence on the sex ratio (male:female). However, it did slightly affect the total number of adult individuals recorded during the sampling months (Fig. 12). Additionally, the study determined that this poison frog species prefers dry leaf litter during the day and green leaves at night (Fig. 13).

**Figure 14:**
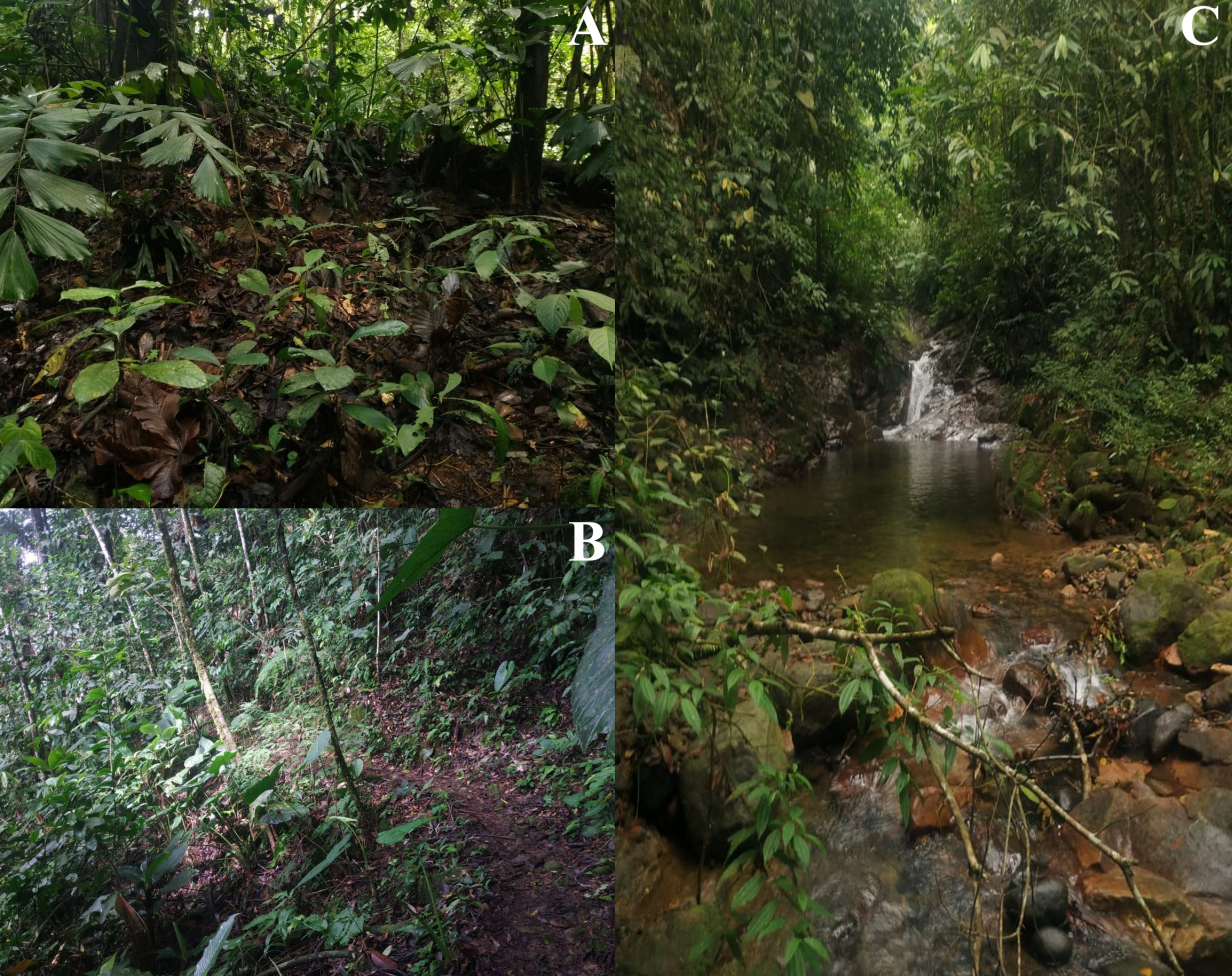
Habitats occupied by *Ameerega ingeri*. (A–B) Secondary forests. (C) Gallery Forest. Photos by Alejandro Navarro-Morales.

**Figure 15:**
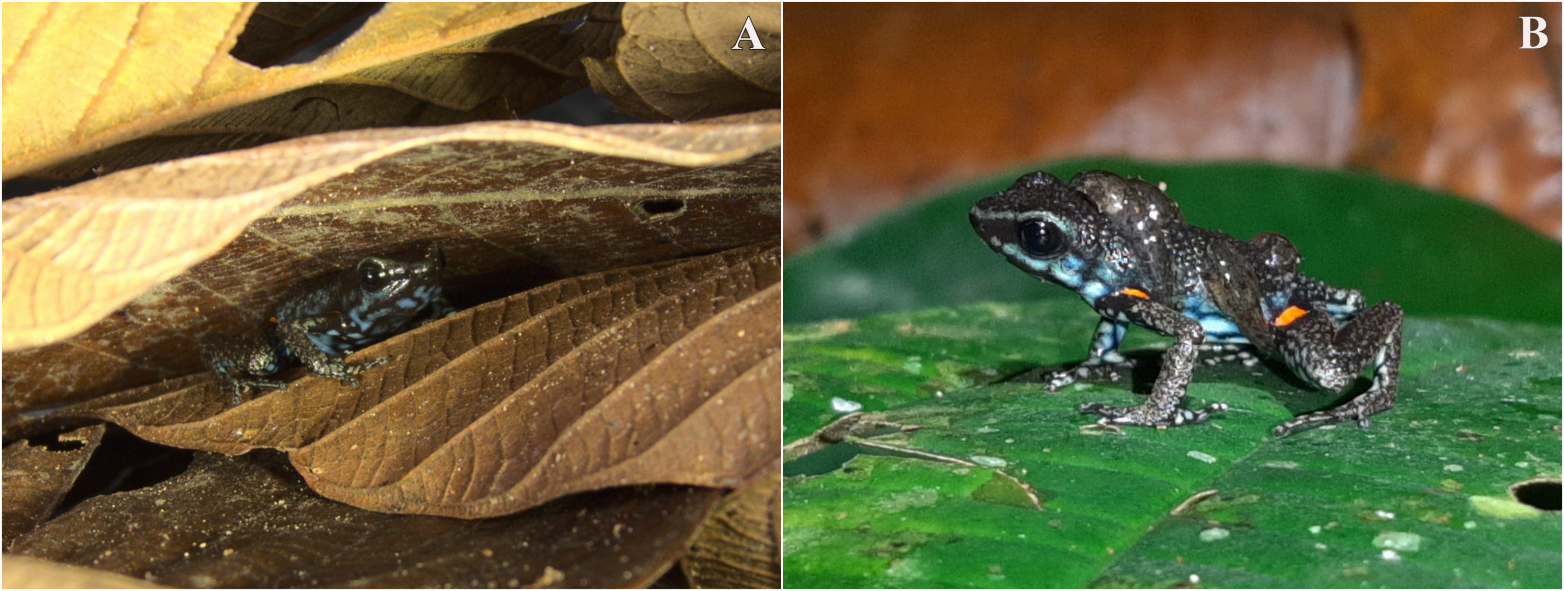
Microhabitat and parental care in *Ameerega ingeri*. Individuals of *Ameerega ingeri:* (A) in leaf litter, (B) male carrying tadpoles on its back. Photos by Yulfreiler Garavito-David.

*Ameerega ingeri* is a relatively common diurnal poison frog inhabiting secondary and gallery forests (Fig. 14). We observed that individuals are primarily active on sunny mornings, moving on the leaf litter. In all localities, over 98% of individuals exhibited a strong preference for leaf litter throughout the daytime (Fig. 15A). Although we also observed inactive individuals on leaves 60–70 cm above the ground, suggesting the use of different plant layers for possible predator avoidance, *A. ingeri* primarily occupies the leaf litter layer. We documented single-parent care for *A. ingeri* by observing a male transporting nine tadpoles on its dorsum at night (Fig. 15B). This event occurred under a plant in wet leaf litter, near a temporary or permanent pond within a secondary forest.

**Figure 16:**
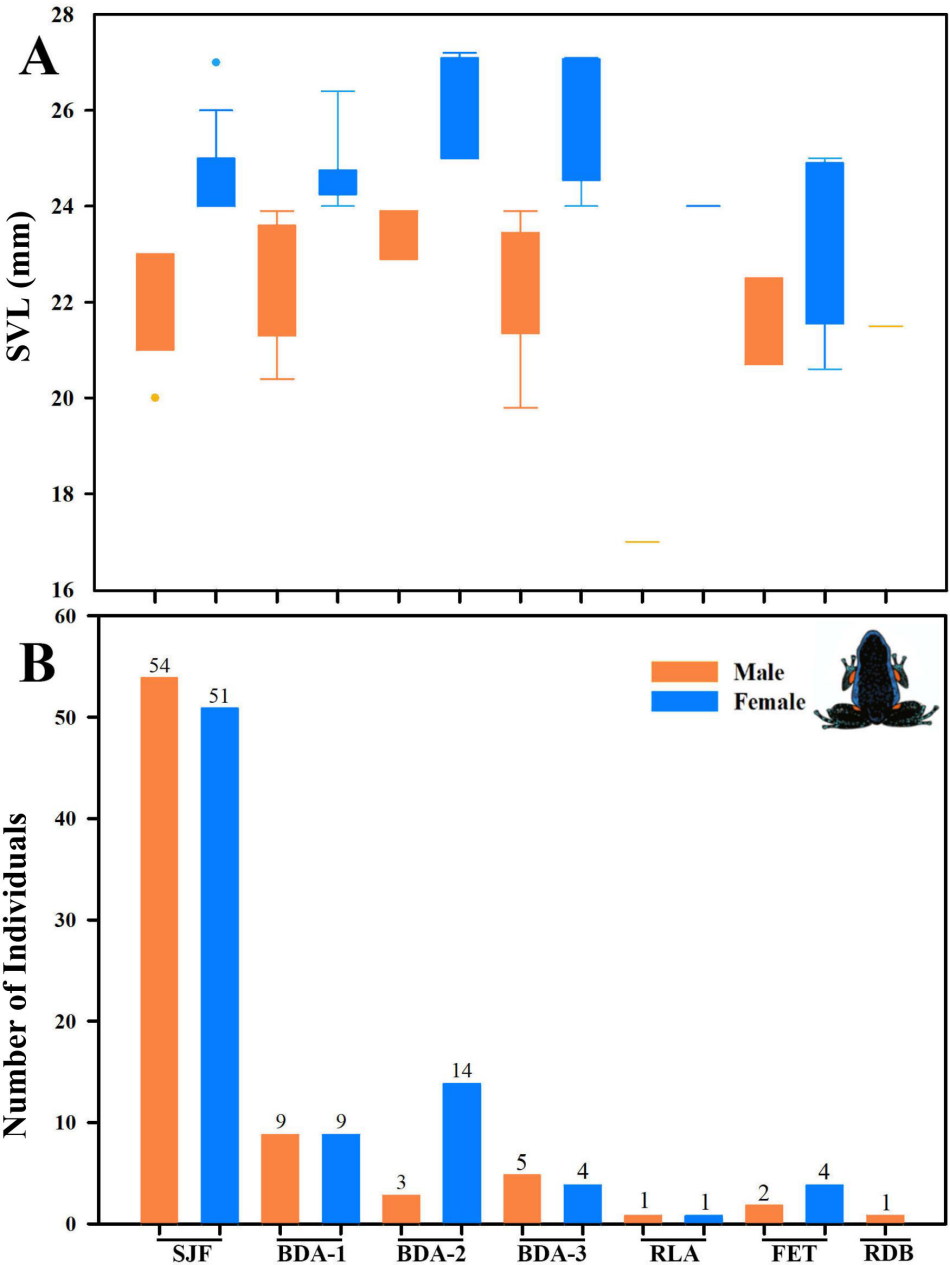
Body size variation and local abundance of *Ameerega ingeri*. (A) Comparison of adult snout-vent-length (SVL) across the seven sampled localities, (B) number of *Ameerega ingeri* individuals recorded per locality (absolute values shown above each bar). Locality codes: SJF = San José del Fragua; BDA-1 = PNN Alto Fragua –Indi Wasi; BDA-2 = Finca El Diviso; BDA-3 = Finca La Esperanza; RLA = Reserva Natural y Ecoturística La Avispa; FET = Finca El Tigre; RDB = Reserva Natural y Agroturística Doña Blanca.

We detected significant differences in body size between males and females of *A. ingeri* within and among localities (Fig. 16A). For instance, females from BDA-2 exhibited pronounced size differences compared to those from SJF, BDA-1, BDA-3, and FET. However, the observed differences among localities might be attributed to small sample sizes (*e.g.*, males and females from other localities with sample sizes similar to SJF). To address this, future studies should expand data collection on these populations. No apparent variability in microhabitat use was observed among individuals. Finally, we recorded a higher number of individuals in San José del Fragua (probably by the high sampling effort in this locality) compared to the other three localities in Caquetá (Fig. 16B).

### Diet

We analyzed nine stomachs, finding food items in four. A total of 53 prey items were identified in four categories (two orders and two families) (Table 4). Among the ants (Formicidae), the genera *Gnamptogenys* and *Strumigenys* were identified as common prey, comprising 44.44% of occurrences and approximately 88.70% of ingested prey, with a high importance index of 74.13%. Hymenoptera (excluding ants) was the second most common prey, constituting 7.54% of ingested prey and having a moderate importance index of 12.39%. Coleoptera (including Curculionidae) were the least common prey, with only two occurrences in total, each recorded in a single stomach, resulting in low representativeness and importance indices of 1.88% and 4.88%, respectively. The calculated niche width was 0.087.

**Table 4:** Diet composition of *Ameerega ingeri* in three locations in the department of Caquetá. (N) Total abundance. (%N) Relative abundance. (PI) Total weight. (%PI) Relative weight. (%OF) Occurrence frequency. (I) Importance index.

Category	N	N%	PI	%PI	OF%	I
Coleoptera	1	1.88	0.0786	1.66	11.11	4.88
Curculionidae	1	1.88	0.0015	1.66	11.11	4.88
Hymenoptera	4	7.54	0.0015	7.40	22.22	12.39
Formicidae	47	88.70	0.0065	89.28	44.44	74.13

### Advertisement call

The advertisement call of *A. ingeri* consists of a series of notes that last 22.0 ± 5.4 s (10.6–30.5), separated by intervals of 62.8 ± 31.0 s (19.1–140.9). Calls have 141.7 ± 28.3 (70.0–192.0) stereotyped tonal notes (non-pulsed) that are emitted in pairs throughout its duration, although the initial 7–12 notes may be emitted individually. Calls have a slight increase in amplitude until 60–90% of their durations, and notes may also exhibit amplitude modulation (up to 50%). These stereotyped tonal notes last 41.0 ± 3.0 ms (16.0–66.0), with the notes in the first part of the call being shorter compared to the others. The silent interval between pairs of notes lasts 170.0 ± 6.0 ms (137.0–415.0), while the interval within pairs of notes is shorter, lasting 49.0 ± 10.0 ms (28.0–71.0 ms). Notes are released at a rate of 386.1 ± 18.2 notes per minute (362.6–413.6); and a rate of 6.4 ± 0.3 notes per second (6.0–6.9).

Calls have a slight increase in frequency until the 56–85% of their durations. Each note has a slight increase in frequency along its duration, an increase of approximately 220 Hz (mean value) from the first to the third quartile frequencies. Dominant frequency peaks are 4,501.3 ± 74.9 Hz (3,876.0–4,866.5); minimum frequency is 4,146.2 ± 31.8 Hz (2,368.7–4,478.9), and the maximum frequency is 5,246.7 ± 692.8 Hz (4,263.6–9,259.3). Each note possesses up to six harmonics. Dominant frequency coincides with the second harmonic.

**Figure 17:**
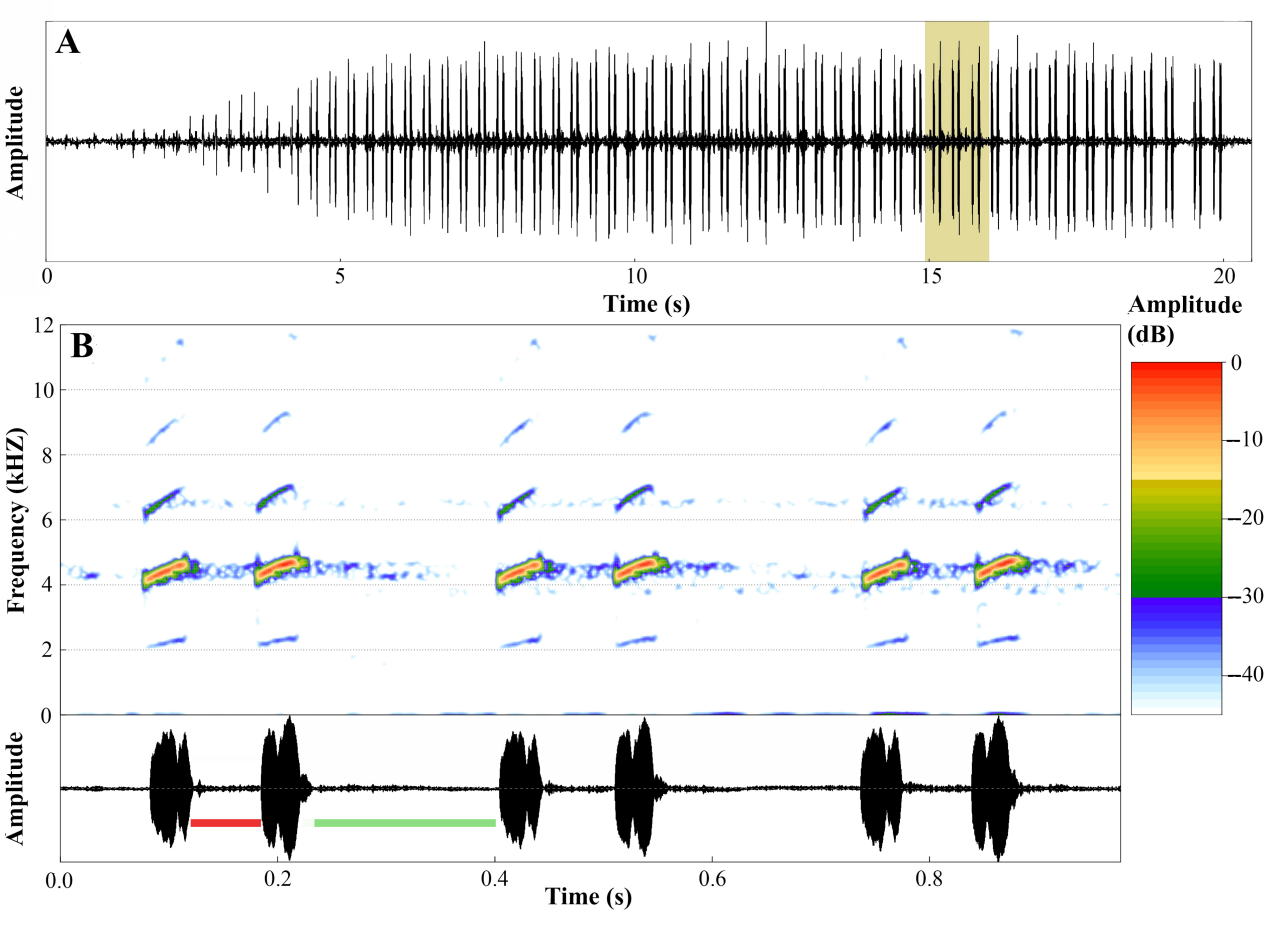
Advertisement call of *Ameerega ingeri*. (A) Oscillogram of the entire advertisement call showing the initial single notes, followed by the stereotyped pairs of notes characteristic of this species, beginning at around 4 s. (B) Audiospectrogram (above) and corresponding oscillogram (below) detailing three pairs of notes highlighted in yellow in A. The red bar represents the interval within pairs of notes, while the green bar represents the interval between pairs of notes. Sound file: TASCAM_0885 A. ingeri 2 individuo. This male (FNJV 123005) was recorded on 26 June 2024 at 05:40–06:30 h.

The fundamental frequency peaks are 2,292.9 ± 13.5 Hz (2,110.3–2,627.1); and the third harmonic frequency range is around 6,599.8 ± 138.1 Hz (6,287.7–7,149.0) Hz. Traits that were classified as static (between-male CV < 11%) were note duration, interval between pairs of notes, notes per minute, notes per second, and all spectral traits. The other traits were classified as dynamic. Values for these quantitative traits of the advertisement call are detailed in Table 5 and spectrograms and oscillograms are depicted in Fig. 17.

**Table 5:** Advertisement call traits of *Ameerega ingeri* based on the recordings of two males (six calls, 850 analyzed notes) from the municipality San José del Fragua locality Caquetá, Colombia. mean ± (SD) Standard deviation range (minimum–maximum). (s) Seconds. (ms) Milliseconds. (Hz) Hertz. (*) 2nd harmonic.

Traits	Values
Call duration (s)	22.0 ± 5.4 (10.6–30.5)
Inter-call interval (s)	62.8 ± 31.0 (19.1–140.9)
Number of notes per call	141.7 ± 28.3 (70.0–192.0)
Note duration (ms)	41.0 ± 3.0 (16.0–66.0)
Inter-note interval (ms)	170.0 ± 6.0 (137.0–415.0)
Interval between pairs of notes (ms)	49.0 ± 10.0 (28.0–71.0)
Notes/minute	386.1 ± 18.2 (362.6–413.6)
Notes/second	6.4 ± 0.3 (6.0–6.9)
Peak of dominant frequency (Hz)*	4,501.3 ± 74.9 (3,876.0–4,866.5)
Min. dominant frequency reached (Hz)*	4,146.2 ± 31.8 (2,368.7–4,478.9)
Max. dominant frequency reached (Hz)*	5,246.7 ± 692.8 (4,263.6–9,259.3)
1st Quartile Frequency*	4,395.7 ± 78.8 (3,789.8–4,608.1)
3rd Quartile Frequency*	4,614.6 ± 84.2 (4,048.2–5,124.9)
Peak of fundamental frequency (Hz)	2,292.9 ± 13.5 (2,110.3–2,627.1)
Peak of 3^**rd**^ harmonic frequency (Hz)	6,599.8 ± 138.1 (6,287.7–7,149.0)

### Molecular data

**Figure 18:**
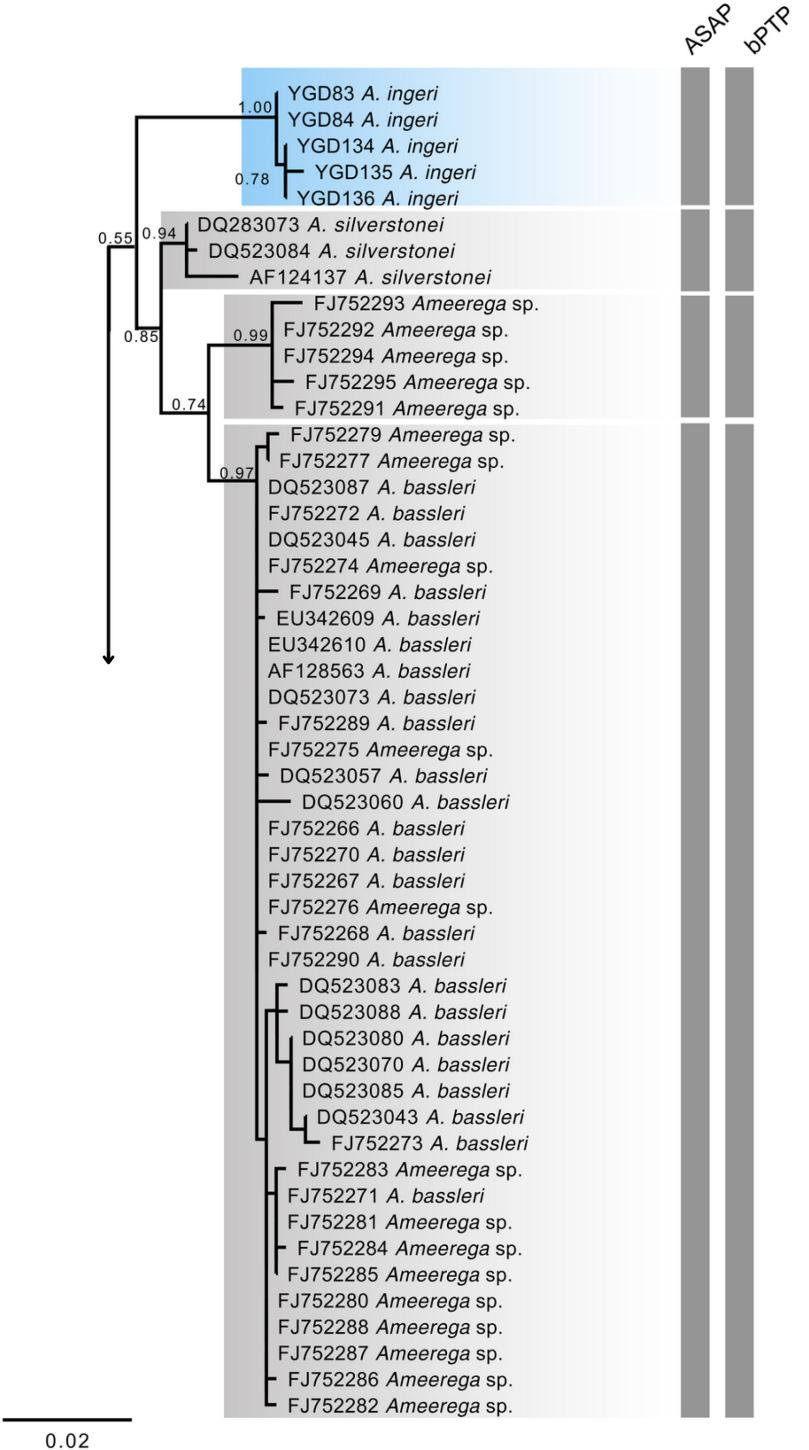
Phylogenetic relationships within the *Ameerega silverstonei* group. Phylogenetic tree based on 16S mitochondrial DNA, showing the evolutionary relationships among species in the *Ameerega silverstonei* group. Bars represent both species’ delimitation method.

The 16S mtDNA gene tree, reconstructed using maximum likelihood (ML) (Fig. 18; Fig. S1), revealed weak support for several deeper nodes. In our investigations, *A. ingeri* was placed within a more exclusive clade (ML 0.60 of bootstrap; BI 0.90 of posterior probability). Within this clade, *A. ingeri* emerged as a sister taxon to *A. silverstonei*, *A. bassleri*, and an unnamed species (*Ameerega* sp.). However, due to the limitations associated with the small fragment of mtDNA employed in this analysis, we exercise caution in drawing definitive conclusions about species relationships. For a comprehensive understanding of *Ameerega* phylogeny, readers are directed to Guillory et al. (2020), where the author extensively discussed the genus’s phylogenetics based on ultraconserved genomic elements and redefined the species groups.

Regarding the uncorrected genetic distance between *A. ingeri* and its congeners, variations ranged from 2.24% (from *A. bilinguis*) to 6.6% (from *A. petersi*). Both species delimitation methods yielded consistent outcomes, with the ASAP method identifying 12 evolutionary entities and the bPTP method recovering 37 evolutionary entities (Fig. 18; Table S2). Both analyses robustly supported the recognition of *A. ingeri* as an independent evolutionary entity.

### Conservation status

Based on available evidence we were able to gather, we propose categorizing *A. ingeri* as Near Threatened (NT) on the IUCN Red List under Criterion B (B1b(iii)+B2b(iii)). Although the estimated Extent of Occurrence (EOO) of ≤20,000 km^2^ and Area of Occupancy (AOO) of 92 km^2^ technically meet the thresholds for the Endangered (EN) category, we consider the available data insufficient to support such an elevated risk level at this time. Nonetheless, the species’ severely limited AOO, along with ongoing declines in both the number of locations and habitat quality (B2b(ii, iii)), suggest that it is close to qualifying as Vulnerable (VU) or even Endangered (EN), and should be closely monitored.

## Discussion

### Taxonomy

It is important to note that this recharacterization of *Ameerega ingeri* is based on newly collected specimens from fieldwork, rather than specifically on the species’ type specimens or topotypes. In fact, the type locality was clarified by Silverstone (1976), but no precise location was established, making it difficult to confidently identify topotypic material. However, we have specimens collected approximately 33 km from the region defined by Silverstone (1976), representing the closest available material to the type locality. We think that the data presented here provide valuable and much-needed clarification on the biology and natural history of this species even if it is not a type material.

The adult specimens examined in this study closely match the holotype description of *A. ingeri* based on four specimens from Caquetá, Colombia (Cochran & Goin, 1970). The original description, which focused exclusively on external morphology, emphasized traits such as granular dorsal skin (except on the snout), a first finger longer than the second on the forelimbs, and a lack of webbing between the toes. While these traits are shared by other poison frog species (Grant et al., 2006; Grant et al., 2017), additional characteristics highlighted by Cochran & Goin (1970), including a distinct, rounded *canthus rostralis,* prominent large eyes, a distinct but relatively small tympanum, a larger tongue, and the apparent absence of external vocal sacs in males, exhibit only slight variations in our specimens. Cochran & Goin (1970) reported a snout-vent length (SVL) of 27.5 mm for *A. ingeri*, although this was a generalized figure that did not consider sexual dimorphism. Our findings reveal clear sexual size differences, with males averaging 21.5 mm and females 23.7 mm SVL.

Cochran & Goin (1970) described the relative lengths of digits and specific hand and toe tubercles. They noted that the first finger is longer than the second and fourth, but shorter than the third, an observation corroborated by our specimens. Regarding tubercles, they mentioned a small oval thumb pad, a larger round palmar callus, and well-developed metacarpal tubercles. Our findings, however, revealed a small inner metacarpal tubercle (thenar) at the base of the first digit, a circular outer metacarpal tubercle (palmar) at the palm’s base, one subarticular tubercle on digits I and II, two on digits III and IV.

Regarding the toes, Cochran & Goin (1970) described long toes with slight webbing at the base and a third toe significantly longer than the fifth, ending in a disk reaching the base of the fourth toe’s antepenultimate phalanx. Our observations align with this description. However, their description of tubercles as a small oval inner metatarsal tubercle, a similarly sized round outer one, and a weak tarsal ridge with a small round tubercle differs from our findings. We observed one subarticular tubercle on digits I and II, two on digits III and V, and three on digit IV. Additionally, there were two metatarsal tubercles, the inner slightly larger than the outer with rounded surfaces.

Cochran & Goin (1970) described preserved specimens of *A. ingeri* as having a slate-black *dorsum*, slightly lighter on the head, with a gray chevron mark before the eyes. Our specimens exhibited a uniformly dark *dorsum*, lacking this pattern. While they reported small pearl-gray spots on the upper proximal femur and axilla, our specimens displayed cream-colored spots in these areas, sometimes extending to the ventral or dorsal surfaces of the calves. The venter was described as slate-black with indistinct light reticulation, differing from our specimens, which showed weakly pronounced brown reticulations and spots, occasionally with two cream spots in the cloacal region.

The coloration of living *A. ingeri* remained unknown until Malambo (2016) and Gutiérrez et al. (2020) provided initial descriptions and photographs. Previous authors (Cochran & Goin, 1970; Jungfer, 1989) suggested a red dorsal coloration similar to *A. bilinguis*, leading to misidentification (Amezquita, Rueda-Almonacid & Rueda-Martínez, 2004). Gutiérrez et al. (2020) reported individuals with orange or yellow spots in axilla, femur, and calves, depending on elevation. Our specimens exhibited a range of orange and yellow spots, regardless of elevation. We hypothesize that yellow coloration might be associated with juvenile stages, given the known color variation in other poison frog species (Brown, 2013). Thus, preliminary observations by CM and MAMO on postmetamorphic development in captivity suggested possible ontogenetic changes in coloration. Examples include *Oophaga pumilio* with 12 color variations (Batista & Köhler, 2008) and tadpole color variations in *A. parvula* and *A. bilinguis* (Poelman et al., 2010).

Tadpole descriptions for *A. ingeri* are currently absent from the literature. However, certain characteristics align with those reported for other *Ameerega* species: an elongate, laterally depressed body, a laterally emarginate oral disc, and W-shaped upper and V-shaped lower jaw sheaths, both slender and finely serrate (Rodríguez & Myers, 1993; Lötters et al., 1997; Duellman, 2005; Twomey & Brown, 2008; Poelman et al., 2010; Sánchez, 2013; Schulze, Jansen & Köhler, 2015; Menin et al., 2017). Notably, stage 25 *A. ingeri* tadpoles exceed the size of sympatric congeners *A. bilinguis, A. hahneli,* and *A. parvula* (Poelman et al., 2010; Menin et al., 2017), as well as other *Ameerega* species such as *A. braccata, A. macero, A. petersi, A. picta, A. rubiventris,* and *A. smaragdina* (Lescure, 1976; Silverstone, 1976; Myers & Daly, 1979; Rodríguez & Myers, 1993; Haddad & Martins, 1994; Lötters et al., 1997).

The oral disc of *A. ingeri* exhibits unique features, including a labial tooth row formula (LTRF) of 2(2)/3 and small marginal papillae, differentiating it from *A. hahneli* (LTRF 1/2 and triangular papillae) (Menin et al., 2017). However, similarities in LTRF (2(2)/3(1)) and small papillae exist with *A. bilinguis, A. flavopicta,* and *A. parvula* (Costa, Facure & Giaretta, 2006; Poelman et al., 2010). *A. ingeri* tadpoles were found on the backs of males near temporary, slow-flowing forest ponds and streams. This reproductive strategy aligns with other *Ameerega* species (Rodríguez & Myers, 1993; Lötters et al., 1997; Poelman et al., 2010; Menin et al., 2017), suggesting a primitive evolutionary state (Grant et al., 2006).

### Distribution

*A. ingeri* was previously considered endemic to Caquetá and Putumayo departments in Colombia (Acosta-Galvis, 2025). The holotype was collected in Aserrío, near the Pescado River, at 260 m elevation, in Valparaíso municipality, Caquetá’s lowlands (Cochran & Goin, 1970). Despite numerous field expeditions, no subsequent collections have been made at the type locality. In fact, Aserrío is not an administrative unit but rather a general term for peasant timber extraction sites (Leon & Restrepo, 2003; Ruiz & Valencia, 2007) in Colombia, and in our case, as it happens along the Pescado River. Although some individuals were found in open habitats, they generally favored areas with cover and low human disturbance, similar to other *Ameerega* species (Twomey & Brown, 2008; Lynch & Arroyo, 2009; Vaz-Silva & Maciel, 2011; Andrade, Victor-Júnior & Vaz-Silva, 2013; Beirne, Burdekin & Whitworth, 2013; Acioli & Neckel-Oliveira, 2014; Serrano-Rojas et al., 2017; Diaz-Ricaurte et al., 2020; Garavito-David, Guevara-Molina & Diaz-Ricaurte, 2020).

However, records of *A. ingeri* in Putumayo (Guerrero-Vargas et al., 2007) suggest that the species may be endemic to Colombian Amazonian foothills. It is unclear whether *A. ingeri* occurs continuously, since there are noticeable gaps in its distribution. These gaps are especially evident within the Colombian Amazon foothills and in relatively well-sampled localities in Caquetá, where it was only found in relatively conserved areas. This species, thus, seems to occur mainly on patches of gallery and secondary forest, differing from its reported association with disturbed areas (Malambo, 2016).

### Diet and natural history

Our study revealed sexual dimorphism in *A. ingeri,* with males smaller than females, a pattern observed in other *Ameerega* species such as *A. altamazonica, A. berohoka,* and *A. trivittata* (Twomey & Brown, 2008; Vaz-Silva & Maciel, 2011; Acioli & Neckel-Oliveira, 2014). To reliably detect such differences, sample sizes typically exceed 30 individuals. While some studies report fewer individuals, limiting statistical power (Forti, Mot & Strüssmann, 2013; Pacheco et al., 2020), our sample size allowed for clear differentiation between sexes.

Crepuscular activity patterns, similar to *A. altamazonica* and *A. shihuemoy* (Twomey & Brown, 2008; Serrano-Rojas et al., 2017), were observed. Ground-dwelling during the day and herbaceous vegetation at night, coupled with male-only parental care, align with the reproductive ecology of other *Ameerega* species (Rodríguez & Myers, 1993; Lötters et al., 1997; Poelman et al., 2010; Menin et al., 2017; Carvajal-Castro et al., 2021).

The predominance of ants in the diet of *A. ingeri* aligns with its ground-dwelling ecology and active foraging strategy, consistent with patterns observed in other dendrobatid frogs such as *Ameerega*, *Epipedobates*, *Oophaga*, and *Ranitomeya* (Biavati, Wiederhecher & Colli, 2004; Darst et al., 2005; Forti et al., 2011; Lima & Eterovick, 2013; Luiz, Contrera & Neckel-Oliveira, 2015; Valderrama-Vernaza, Ramírez-Pinilla & Serrano-Cardozo, 2009; Pacheco et al., 2020). The identification of predatory leaf-litter ant genera, such as those reported by Lattke et al. (2008) and Booher & Hoenle (2021), further supports this association with microhabitats rich in invertebrate diversity The occasional presence of non-ant prey, such as beetles and parasitic microwasps, may indicate that *A. ingeri* includes a broader range of small arthropods in its diet than previously assumed. Alternatively, these items might be consumed opportunistically due to similarity in size and movement to ants. Further data would be needed to evaluate the consistency and relevance of these prey types.

### Acoustic communication, differences and challenges

The temporal and spectral traits of *A. ingeri*’s advertisement call easily distinguish it from all other congeneric species with known calls. Thus, acoustic data serve as a reliable source for identifying and diagnosing this species. The call can be particularly useful when there is high morphological similarity among species or when molecular analyses are not readily accessible. We strongly recommend the extensive use of the species’ call whenever possible to avoid potential identification errors in the future.

The species with a call most similar to *A. ingeri* is *A. bilinguis*. They can be differentiated based on the silent interval within their pairs of emitted notes. Both *A. ingeri* and *A. bilinguis* are basal species in the phylogeny (Guillory et al., 2020), exhibiting call patterns that are quite distinct from other congeneric species. This difference may represent a call pattern of some ancestors of many *Ameerega* species.

A broader approach to understanding the evolution of acoustic traits in *Ameerega* species is greatly needed. Although no species of this genus was included, a study evaluating the differential evolution of acoustic traits in dart-poison frogs (Dendrobatidae) has already been conducted and yielded very interesting findings (Erdtmann & Amézquita, 2009). We endorse future studies to thoroughly examine the evolution of advertisement calls in *Ameerega* species using appropriate methods and databases.

It is also important to highlight the pressing necessity for recharacterizing the calls of most species in the genus, as many studies are based on small samples or are very brief and lack detail in their acoustic descriptions. Modern recorders, microphones, and softwares will greatly assist in providing a better and more detailed characterization of this crucial dataset for the taxonomy and ecology of these frogs.

### Phylogenetic relationships

The phylogenetic analysis places *A. ingeri* as a well-supported, distinct lineage, with high confidence values (1.00, 0.78) separating it from *A. silverstonei* and *A. bassleri*. Besides, both delimitation methods found it as an independent lineage. Despite the limitations of 16S mitochondrial DNA (mtDNA) for building deep phylogenies, especially in genera like *Ameerega*, which exhibit rapid radiations (Guillory et al., 2020) and short internodes that reduce the resolution of mitochondrial markers, this marker remains useful for species identification and delimitation (Koroiva & Santana, 2022), as demonstrated in this analysis. The clustering of *A. ingeri* with high bootstrap values supports its status as a distinct species, underscoring the utility of 16S mtDNA for identifying lineages, even though it may not fully capture the complex evolutionary relationships within *Ameerega*. To better understand the phylogenetic position of *A. ingeri*, Guillory et al. (2020) conducted a more comprehensive study using genomic data. Their findings consistently recovered *A. ingeri* as sister to a clade containing all other *Ameerega* species, except *A. silverstonei*. They support that *A. ingeri* lacks close relatives within the genus, potentially representing a lineage from *Ameerega*’s early evolutionary expansion. While mitochondrial data provides insights into species boundaries (Vences et al., 2012), genomic data is crucial for elucidating deeper evolutionary histories, as seen in Guillory et al. (2020). Their study emphasizes that *A. ingeri* may have diverged early in the genus’ history, explaining its unique position within the phylogeny and suggesting it may be one of the oldest surviving lineages of *Ameerega*.

### Knowledge gaps and conservation challenges

For a long time, *A. ingeri* remained poorly understood, with limited information of its morphology, distribution, natural history, bioacoustics, molecular characteristics, and conservation status. This lack of data is particularly concerning given the species’ fluctuating conservation assessments, which have ranged from Critically Endangered (CR) (Amezquita, Rueda-Almonacid & Rueda-Martínez, 2004) to Vulnerable (VU) (Malambo, 2016) and Data Deficient (DD) (IUCN SSC Amphibian Specialist Group, 2017).

While our localized fieldwork suggests that *A. ingeri* populations in the specific localities surveyed within the Caquetá department may exhibit short-term stability, we acknowledge that rapid deforestation across the broader region (Botero & Rojas, 2018) represents a significant threat to the long-term viability of these populations across their entire distribution (Vieira et al., 2008). The low recapture rate observed may point to a large population size, but could also be influenced by factors such as short lifespan, high mobility, or low detection probability. Therefore, any inference about population stability should be interpreted with caution and supported by long-term monitoring efforts. The ongoing habitat loss, coupled with our estimated EOO and AOH, aligns with the IUCN’s categorization of the species as Near Threatened (NT), primarily due to habitat degradation and fragmentation. This decline is primarily driven by expanding cattle ranching, deforestation, wildfires (particularly bushfires), agricultural encroachment, and illegal mining—all widely reported in the region (Botero & Rojas, 2018). Importantly, in every surveyed location, the species was found mainly within vegetated areas. This means the high number of fragments and the low connectivity between them present a significant future challenge for the species’ survival. Even though *A. ingeri* might appear to have a moderate distribution in the ecoregion, constant population monitoring and more comprehensive studies on its population ecology are urgently needed. This is due to the low connectivity between remaining populations, which is exacerbated by severe habitat fragmentation and alteration of the landscape matrix, making the species considerably more vulnerable than current data might suggest. The cumulative impact of these threats implies that an Endangered (EN) classification may ultimately be necessary, underscoring the urgent need for monitoring its populations to fully assess these ongoing pressures.

Despite existing environmental policies, deforestation continues, exacerbated by projects like the La marginal de la Selva’ road, which directly impact known *A. ingeri* habitats. Furthermore, our field studies revealed illegal coca cultivation within the protected PNN Alto Fragua-Indi Wasi, highlighting the immense challenges of biodiversity conservation amidst ongoing conflict and instability following FARC demobilization. We acknowledge that a comprehensive assessment of the species’ overall population dynamics and stability requires further research across its entire range.

This study underscores the critical need for detailed species descriptions, including distribution, conservation status, and natural history, especially within the context of broader environmental challenges. Such comprehensive data is vital for developing effective management and conservation strategies for *A. ingeri* and other threatened amphibians in the region.

## Supplemental Information

10.7717/peerj.20078/supp-1 Supplemental Information 1 Interspecific phylogenetic relationships of *Ameerega silverstonei* group from a maximum likelihood cladogram inferred from the mitochondrial sequences using Bayesian analysis

10.7717/peerj.20078/supp-2 Supplemental Information 2 New sequence data of *Ameerega ingeri*

10.7717/peerj.20078/supp-3 Supplemental Information 3 Newick tree file of 16S mtDNA sequences for Ameerega ingeri and all congeneric species NWK files can be opened and visualized in commonly used software such as FigTree, MEGA, or in R with packages like ‘ape’ or ‘phytools’.

10.7717/peerj.20078/supp-4 Supplemental Information 4 Distribution records of *Ameerega ingeri* over time in Colombia and Ecuador; GenBank accession numbers for the sequences of the *Ameerega silverstonei* group used in this study; morphological measurements of adult specimens collected of specimens collected of [i] A.

## Acronyms

NMNH: Smithsonian National Museum of Natural History in Washington, D.C., United States
UAM - H: Colección de Anfibios del Museo de Historia Natural de la Universidad de la Amazonia, Florencia-Caquetá, Colombia
MHNUC: Museo de Historia Natural de la Universidad del Cauca, Cauca, Colombia

## Appendix I

**Specimens examined**

*Ameerega ingeri*. –COLOMBIA: CAQUETÁ, Valparaiso, AserRío Rio Pescado (type locality): USNM 146846–9; Belén de los Andaquíes, Resguardo Indígena La Cerinda: UAM-H 2055–6; Aletones: UAM-H 2060; Parque Natural Municipal La Resaca: UAM-H 2061–3; Tendidos: UAM-H 2066; Finca El Diviso: UAM-H 2074–6; Finca La Esperanza: UAM-H 2077–9; Parque Nacional Natural Alto Fragua-Indi Wasi: UAM-H 2080–1; El Paujil, Reserva Natural y Agroturistica Doña Blanca: UAM-H 2049; Florencia, Reserva Natural y Ecoturistica La Avispa: UAM-H 1652, 2048; Finca El Tigre: UAM-H 1653, 2047, 2050–2, 2054; Finca El Ceilan: UAM-H 2064–5; Finca Sucre: UAM-H 2067–8. San Jose del Fragua, Finca Don Neder: UAM-H 2057–9; Vereda Cafetales: UAM-H 2069–73; Finca El Chingaral: UAM-H 2082–4. PUTUMAYO, Mocoa, Umbria: USNM 147223; Puerto Limon: MHNUC He An 000476.

## Additional Information and Declarations

### DNA Deposition

The following information was supplied regarding the deposition of DNA sequences:
PV637188, PV637189, PV637190, PV637191, PV637192.

### Data Availability

The following information was supplied regarding data availability: The raw data are available in the Supplementary Files.
