# Supplementary figures and images for: Revisiting the Poison Dart Frog Ameerega ingeri (Anura: Dendrobatidae): external morphology, tadpoles, natural history, distribution, advertisement call, phylogenetic position and conservation
Source: PeerJ. 2025 Dec 12;13:e20078. doi: 10.7717/peerj.20078 (PMC12704335; doi:10.7717/peerj.20078)

Silverstoneia\_nubicola\_DQ502161

Colostethus\_pratti\_DQ502163

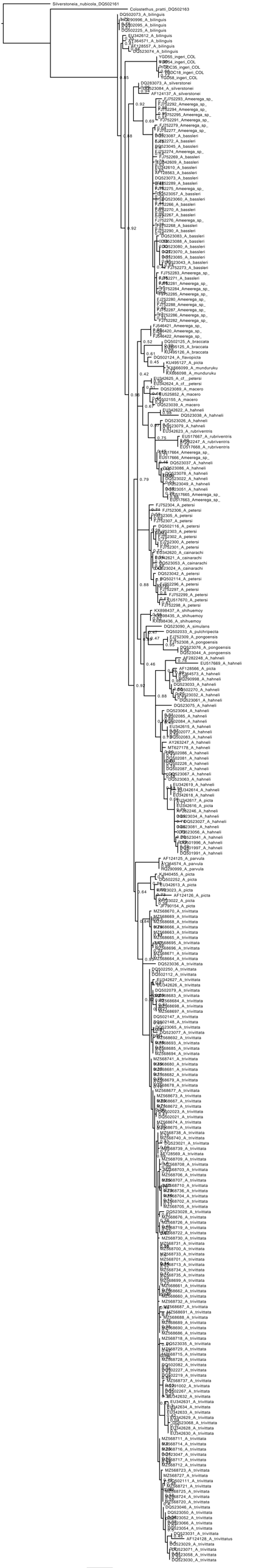

Supplement: Supplemental Information 1 [file peerj-13-20078-s001.pdf]
